# Electrochemical simulation of psychotropic drug metabolism compared to *in vivo* processes using liquid chromatography and mass spectrometry

**DOI:** 10.3389/fphar.2025.1637852

**Published:** 2025-08-28

**Authors:** Paulina Jerszyńska, Małgorzata Szultka-Młyńska

**Affiliations:** ^1^ Department of Environmental Chemistry and Bioanalytics, Faculty of Chemistry, Nicolaus Copernicus University, Torun, Poland; ^2^ Centre for Modern Interdisciplinary Technologies, Nicolaus Copernicus University, Torun, Poland

**Keywords:** electrochemistry, *in vitro*, *in vivo*, liquid chromatography, mass spectrometry, psychotropic drugs

## Abstract

**Introduction:**

Psychotropic drugs strongly affect the human psyche through their ability to modulate the neurotransmitter activity and to treat mental disorders and diseases. Monitoring of psychotropic drugs in clinical studies is significant. Thus. establishing methodologies for analyzing these drugs and their pharmacologically active metabolites in biological matrices is essential for patients’ safety. Therefore, therapeutic drug monitoring (TDM) of these drugs in patients receiving pharmacotherapy in psychiatric hospitals is necessary to avoid medical complications, psychiatric adverse effects, or poisoning. In addition to TDM, the main factor in pharmacokinetics that should be monitored along with the drug is its metabolic pathway. The literature on transformation products (TPs) resulting from the psychotropic drug degradation is limited. Hence, to investigate the potential TPs of target compounds, electrochemistry (EC) and liver microsome assays were used to generate TPs, which were further characterized using liquid chromatography-tandem mass spectrometry (LC-MS/MS). The results obtained by EC-(LC)-MS and liver microsome assays were compared with conventional *in vivo* studies by analyzing biological samples (human plasma) from patients.

**Methods:**

The electrochemical mimicry of the oxidative phase I and II metabolism was achieved in a thin-layer cell equipped with a boron-doped diamond (BDD) working electrode under controlled potential conditions. Structures were proposed for the electrochemically generated products based on the MS/MS experiments. Moreover, in order to examine the proposed metabolic pathways of target compounds, the incubation with human liver microsomes was applied. Additionally, a sensitive, specific, and rapid LC-MS/MS method was developed and validated to quantify selected drugs and their metabolites in biological samples. The preparation of biological samples was accomplished through microextraction by a packed sorbent (MEPS). Finally, the results from LC-MS/MS analysis of biological samples, liver microsomes and electrochemical TPs were compared to evaluate the quality of electrochemical metabolism mimicry.

**Results and discussion:**

Data from *in vivo* experiments agreed with the data from electrochemical oxidation, which predicted some of the potential metabolites found in the human liver microsomes. EC–(LC)-MS is well-suited for the simulation of the oxidative metabolism of selected psychotropic drugs and acts as the orthogonal source of information about drug metabolites compared to liver microsomes and biological matrices. EC-(LC)-MS enables the direct identification of reactive TPs, circumvents time-consuming sample preparation and is ethically advantageous because it reduces the need for animal experiments.

## 1 Introduction

**FIGURE 1:**
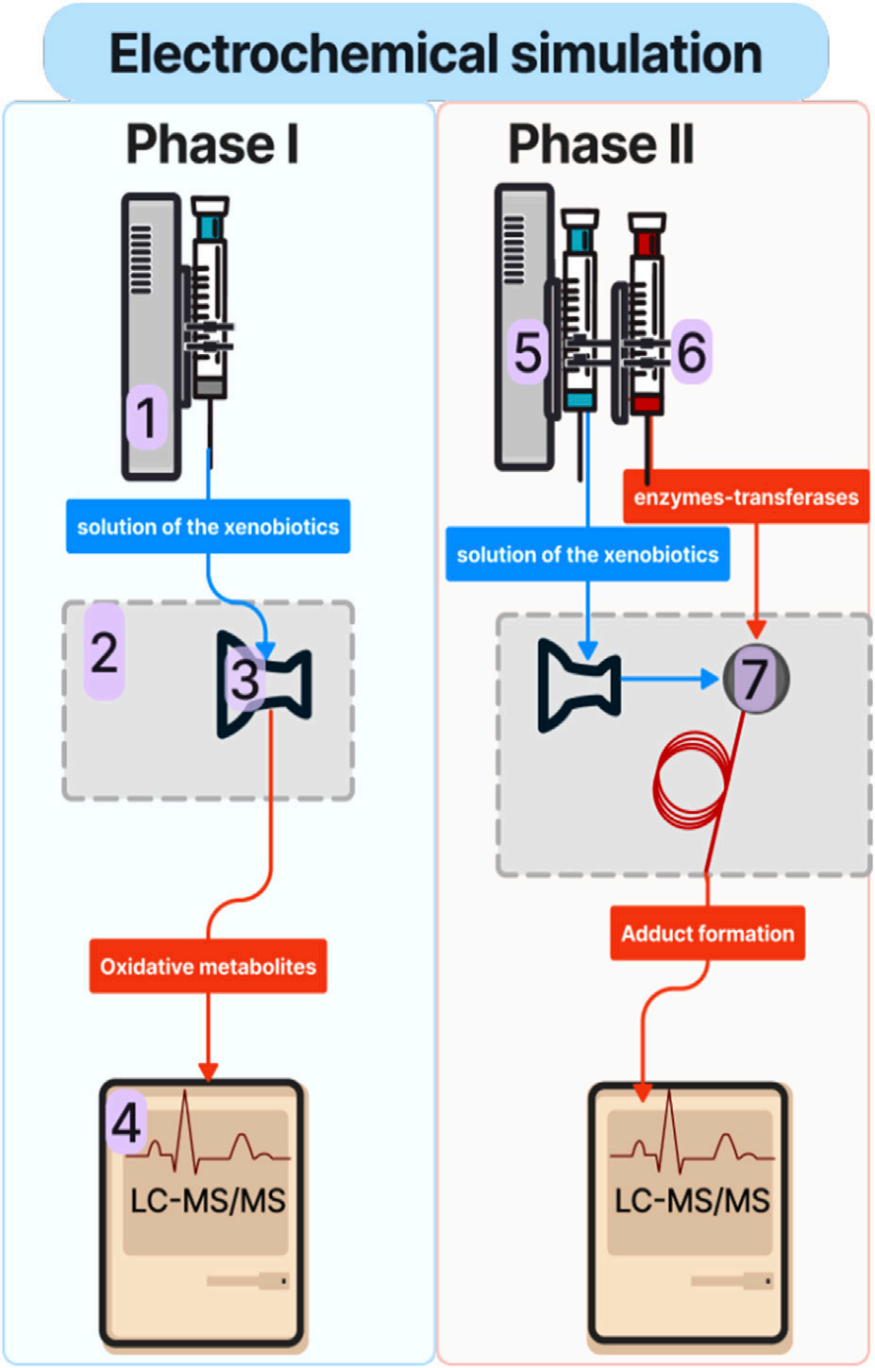
Schematic of the experimental EC-LC-MS/MS setup for phase I and phase II metabolic simulation studies: 1: syringe pump; 2: electrochemical reactor; 3: electrochemical cell; 4: liquid chromatography coupled with tandem spectrometry mass (LC-MS/MS), 5: syringe containing a solution of xenobiotic; 6: syringe containing a solution of phase II conjugation agent (e.g., GSH); 7: reaction coil.

Psychotropic drugs strongly impact the human psyche by affecting the neurotransmitter action and by treating mental disorders and diseases (Tuzimski and Petruczynik, 2020). Unfortunately, only a few aspects of the mechanism of action of psychotropic drugs are known. Therefore, therapeutic drug monitoring (TDM) of these drugs in patients receiving pharmacotherapy in psychiatric hospitals is necessary to avoid medical complications, poisoning, or non-response reactions. Performing TDM requires highly sensitive methods to detect low concentrations of drugs [μg/L - mg/L] in small volumes of biological samples (blood, saliva) and ensure accuracy and specificity. The reference method in TDM is high-performance liquid chromatography (HPLC) or ultra-high-performance liquid chromatography (UHPLC) coupled with tandem spectrometry mass (MS/MS). This technique provides high selectivity and sensitivity. In addition, it enables the analysis of a wide range of analytes in a short period. Including TDM, the main factor in pharmacokinetics that should be monitored along with the drug is its metabolic pathway, related to the formation of metabolites (Tuzimski and Petruczynik, 2020; Kang and Lee, 2009). Drug metabolism is governed by metabolic enzymes (e.g., cytochrome P450). These enzymes show especially high activity in the liver, rendering it the main organ in the drug metabolism. There are two pathways of drug metabolism, called phase I and phase II. Phase I comprises non-synthetic reactions involving the compound’s oxidation, reduction, and hydrolysis. Phase II is described as conjugation reactions, which include conjugating the drug itself or its phase I metabolite with an endogenous compound, generally leading to the formation of less toxic products. Conjugation reactions occur with the participation of specific enzymes-transferases (e.g., sulfotransferases (SULT), glycine, cysteine, glutathione (GSH) transferase, thiol or thiopurine methyltransferase (TMT, TPMT)). Metabolites formed during phase I and II can significantly affect the human body. However, some products of phase I can have a short half-live and quickly form conjugates with proteins and other endogenous macromolecules. Therefore, determining reactive metabolites in plasma or whole blood can be difficult and sometimes even impossible (Kang and Lee, 2009). Conventional methods of metabolite studies are *in vitro* and *in vivo* tests. Both approaches involve the use of enzymes, tissues or whole organs (*in vitro*) or organisms (*in vivo*). Both methods have advantages and disadvantages. Drug metabolism *in vitro* tests, like microsomes and hepatocytes, reflect the metabolic processes in the organ from which they originate, mainly the liver. *In vitro* and *in vivo* differences often result from involvement of other organs, or from different kinetics (like secondary metabolite formation due to long incubation times), etc. However, *in vivo* tests are critical with regards to animal well-beings and generate ethical considerations. Thus, their number should be limited considerably and refined to provide the most valuable information while keeping their application to a minimum. In addition, to *in vivo* and *in vitro* approaches, the combination of electrochemistry (EC) and mass spectrometry (MS) was investigated as a purely instrumental approach to simulate oxidative and reductive phase I metabolic reactions and reduce the number of animal studies (Telgmann et al., 2012; Lohmann et al., 2010). A solution of xenobiotics is transferred through an electrochemical cell, where redox reactions occur under controlled conditions. A direct *online* connection to an LC system can be used to separate the electrochemical transformation products before the mass spectrometric detection. The most significant advantage of this technique is the ability to thoroughly investigate potential phase I of metabolites, in a controlled and simple matrix compared to *in vitro* and *in vivo* systems. In addition, selected phase II reactions can also be simulated by addition of conjugation agents such as GSH to the electrochemical cell effluent (Telgmann et al., 2012; Lohmann et al., 2010; Lu et al., 2012). A schematic experimental setup of the electrochemical metabolism simulation is depicted in Figure 1.

Numerous literature reports indicate that EC-MS is used to simulate the metabolism of drugs such as clonazepam, chlorpromazine (Lu et al., 2012), clozapine (van Leeuwen et al., 2005), tetrazepam (Baumann et al., 2009), thyroxine (Mak et al., 2015), fesoterodine (Kučerová et al., 2015), methimazole (Chipiso and Simoyi, 2016), and lidocaine (Nouri-Nigjeh et al., 2010). Although the number of publications is increasing, the application of EC-LC-MS/MS has not been established yet for the routine analysis outside academic research. The results of electrochemical metabolism mimicry need to be compared with those obtained by *in vitro* methods to introduce the method in life sciences responsibly. Still, EC-MS is a promising technique for quantitative and qualitative analysis that allows simulations of the metabolism of drugs, peptides, proteins, or DNA in living organisms (Nováková and Vlčková, 2009). As can be seen, the EC/MS is most often used in the study of drug metabolism. Many compounds in the human body are oxidized or reduced because they have redox properties (over 90%). Oxidation reactions, involving the transfer of electrons, play a crucial role in the metabolism of drugs, influencing their efficacy, toxicity, and overall therapeutic outcome. These reactions, primarily catalyzed by enzymes like cytochrome P450, can transform drugs into metabolites that are more easily excreted or activate or deactivate a drug. Understanding the role of oxidation reactions in drug metabolism is essential for designing effective and safe drugs. Electrochemistry has been used in biomedical chemistry for years to study the oxidation-reduction properties of pharmaceuticals. Combining electrochemistry with mass spectrometry EC/MS allows for generating and identifying electrochemical products for biologically active compounds with diverse physicochemical properties. Additionally, the introduction of various modifications, including the combination of EC/MS with separation methods (liquid chromatography LC and capillary electrophoresis CE) in *online* or *offline* mode, increases the efficiency of the degradation process, shortens the time between the production and detection of a given metabolite (electrochemical product), which is associated with the identification of reactive compounds with a short lifetime (Bussy et al., 2015; Karst, 2004; Permentier et al., 2008). This method is classified as an instrumental technique in which biological systems are not used. It allows for identifying end products and intermediate electrochemical reactions of the tested compounds. One of its advantages is identifying oxidation products immediately after their formation. This approach can effectively predict the oxidation process initiated by single-electron oxidation, such as *N*-dealkylation, *S*-oxidation, *P*-oxidation, the oxidation of alcohols, the hydroxylation of aromatic systems, and dehydrogenation. Electrochemistry is not advantageous for reactions initiated by direct hydrogen atom acquisition, such as O-dealkylation or aliphatic hydroxylation of unsubstituted aromatic rings, due to the too-high oxidation potential required for the electrochemical oxidation reaction (Bussy et al., 2015; Karst, 2004; Permentier et al., 2008).

The primary objective of the research is to compare electrochemical transformation products to potential metabolites of selected psychotropic drugs in biological samples ranging from liver microsomal fraction incubations to human samples.

## 2 Materials and methods

### 2.1 Chemicals

Standards of drugs (Table 1) and their metabolites: quetiapine, 7-hydroxyquetiapine, norquetiapine, clozapine, clozapine-*N*-oxide, *N*-desmethylclozapine, aripiprazole, dehydroaripiprazole, risperidone, 9-hydroxyrisperidone, citalopram, *N*-desmethylcitalopram, venlafaxine, *O*-desmethylvenlafaxine, vilazodone, M10 metabolite, vortioxetine, levomepromazine, perazine, olanzapine, and *N*-desmethylolanzapine were obtained from Sigma-Aldrich Chemie (Steinheim, Germany). Water (99.9%), methanol, acetonitrile, ammonium acetate, ammonium formate, ammonia, and formic acid (all LC-MS purity) were supplied by Sigma-Aldrich Chemie (Steinheim, Germany). Isopropanol (99.9%) was purchased from J.T. Baker (Deventer, Netherlands). Water was obtained from Milli-Q deionized water (MiliporeIntertech, Bedford, USA). Methanol pure p. a. (POCH S.A., Gliwice, Poland) was used to clean the EC system. GSH (LC-MS grade, Sigma-Aldrich Chemie, Steinheim, Germany) was used for phase II conjugation reactions. Human liver enzymes of the microsomal fraction (HLMs) (Sigma-Aldrich Chemie, Steinheim, Germany), nicotinamide adenine dinucleotide β-phosphate (β-NADPH) tetrasodium salt (Sigma-Aldrich Chemie, Steinheim, Germany), magnesium chloride hexahydrate (MgCl_2_×6H_2_O), potassium chloride (KCl), potassium diphosphate (KH_2_PO_4_), ethylenediaminetetraacetic acid (EDTA) (Sigma-Aldrich Chemie, Steinheim, Germany) were used for *in vitro* approaches.

**TABLE 1:** Studied psychotropic drugs and their properties.

Psychotropic drug	Abbreviation	Chemical structure	Chemical formula	Molar mass [g/mol]	pKa	Applications
Quetiapine	*QUE*	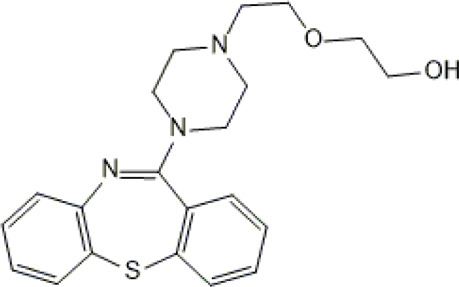	C_21_H_25_N_3_O_2_S	383.51	pKa_1_ = 7.06 pKa_2_ = 15.12	Schizophrenia, manic episodes, delusions, bipolar, depression
Clozapine	*CLO*	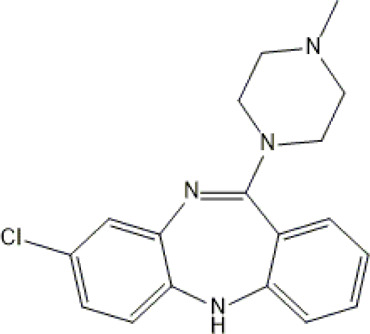	C_18_H_19_ClN_4_	326.82	7.5	Schizophrenia
Aripiprazole	*ARI*	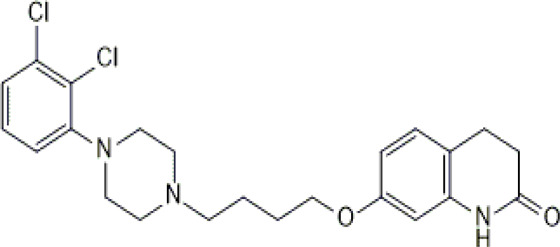	C_23_H_27_Cl_2_N_3_O_2_	448.39	pKa_1_ = 7.46 pKa_2_ = 13.51	Schizophrenia, bipolar affective disorder and manic effects
Risperidone	*RIS*	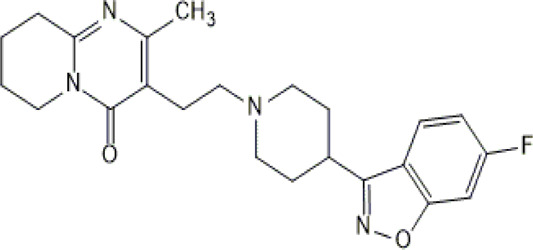	C_23_H_27_FN_4_O_2_	410.49	8.76	Schizophrenia, manic episodes. Treatment of aggression in patients with Alzheimer’s disease. Treatment of aggression in conduct disorder in children
Citalopram	*CIT*	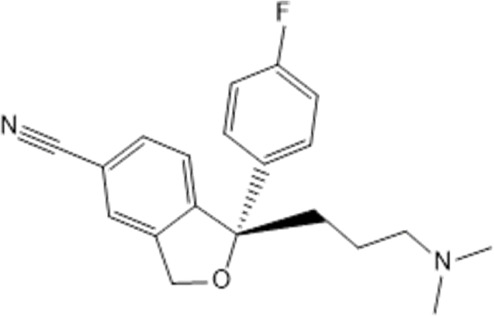	C_20_H_21_FN_2_O	324.39	9.78	Depression. Relapse prevention of recurrent depressive disorder
Venlafaxine	*VEN*	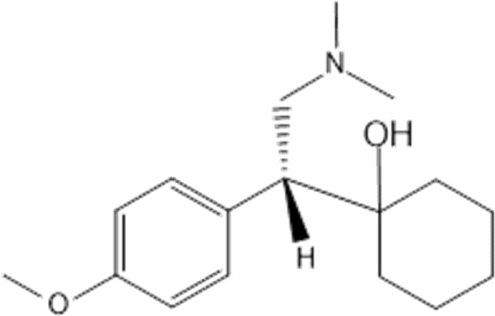	C_17_H_27_NO_2_	277.40	pKa_1_ = 8.91 pKa_2_ = 14.42	Depression, including depressive disorders with anxiety. Generalized anxiety disorder. Social phobia. Paroxysmal anxiety syndrome with or without agoraphobia. Prevention of recurrence of depression
Vilazodone	*WIL*	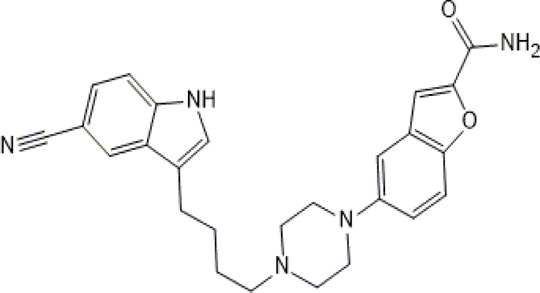	C_26_H_27_N_5_O_2_	441.52	7.1	Depression, including depressive disorders with anxiety
Vortioxetine	*VOR*	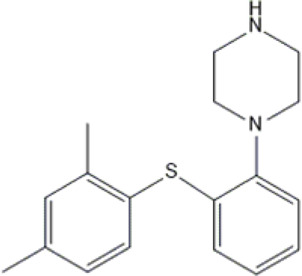	C_18_H_22_N_2_S	298.45	8.85	Depression
Levomepromazine	*LEV*	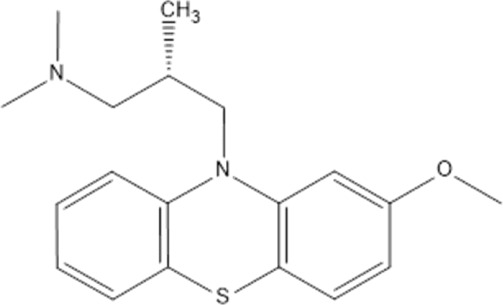	C_19_H_24_N_2_OS	328.47	9.19	Mental illnesses with symptoms of motor and psychomotor agitation, paranoid syndromes, schizophrenia. Symptoms associated with epilepsy, mental retardation, depression with anxiety
Perazine	*PER*	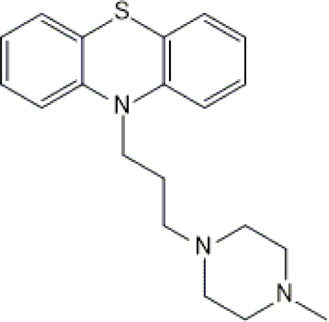	C_20_H_25_N_3_S	339.50	8.41	Schizophrenia and acute psychotic disorders with symptoms of psychomotor agitation, mania and delusions
Olanzapine	*OLA*	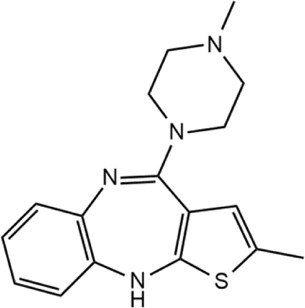	C_17_H_20_N_4_S	312.44	pKa_1_ = 7.24 pKa_2_ = 14.17	Schizophrenia. bipolar affective disorder

### 2.2 *In silico* metabolite prediction

Putative metabolites of the target pharmaceuticals were predicted using selected compounds’ SMILES strings.

(QUE: N\1 = C(\c3c(Sc2c/1cccc2)cccc3)N4CCN(CCOCCO)CC4;
CLO: CN1CCN(CC1)C2 = Nc3cc(ccc3Nc4c2cccc4)Cl;
ARI: Clc4cccc(N3CCN(CCCCOc2ccc1c(NC(=O)CC1)c2)CC3)c4Cl;
VEN: OC2(C(c1ccc(OC)cc1)CN(C)C)CCCCC2);
VOR: CC(C=C(C)C=C1) = C1SC2 = C(N3CCNCC3)C=CC = C2).

Through the free online software GLORYx (de Bruyn Kops et al., 2021) and Biotransformer 3.0 (Djoumbou-Feunang et al., 2019). Briefly, GLORYx integrates machine learning-based site of metabolism (SoM) prediction with biotransformation reaction rule sets to predict and classify the putative structures of metabolites that could be formed by phase I and/or phase II metabolism. Biotransformer 3.0 is an open-access software tool that supports the rapid, accurate, and comprehensive prediction of small molecules’ metabolism in mammals and environmental microorganisms. ChemDraw software generated all metabolite structures (PerkinElmer, Inc., Waltham, MA, USA).

### 2.3 EC-MS/MS conditions

Electrochemical experiments were performed with a ROXY™ system (Antec, Alphen aan den Rijn, Netherlands) containing a potentiostat, an oven compartment, and an infusion pump. A ReactorCell™ (Antec, Alphen aan den Rijn, Netherlands) was employed for the electrochemical transformation. The ROXY™ system was controlled *via* the Dialogue™ software (Antec, Alphen aan den Rijn, Netherlands). Oxidation products were directly transferred to the electrospray ionization (ESI) source of the mass spectrometer (Agilent, Waldbronn, Germany), which was operated in positive ion mode. The MassHunter software (Agilent, Waldbronn, Germany) was used to controlthe mass spectrometer and data analysis. The experiments consisted of two stages: electrochemical oxidation for phase I-like transformation and the addition of GSH for phase II-like conjunction. To carry out the first stage of experiments, 5 and 10 μM solutions of the drug in the selected mobile phase were prepared. At a flow rate of 10 μL/min, the solution of the target compound was passed through the electrochemical cell heated to 36,7 °C with selected electrode by a syringe pump. The electrochemical cell was coupled online and offline to the mass spectrometer. The recorded datawere obtained by increasing the working electrode potential linearly from 0 to 2000 mV (for glassy carbon (GC), gold (Au), and platinum (Pt) electrodes) or from 0 to 3,000 mV (for the BDD electrode) at a rate of 10 mV/s (Supplementary Table S1). For phase II-like conjugation reactions, the cell effluent was mixed with a GSH-solution in the same mobile phase as the analyzed drug in a reactor coil. The effluent of the reaction coil was transferred online and offline to the mass spectrometer.

### 2.4 LC-MS/MS conditions

Operating parameters of the triple quadrupole mass spectrometer with an ESI interface operating in positive ion mode for the analyzed drugs and their metabolites, biological samples, and *in vitro* tests were carried out on LC-ESI-MS/MS 8050 (Shimadzu, Tokyo, Japan). LC measurements were carried out using an ACE C18 column (150 mm × 4.6 mm) with mobile phase A (water with 0.1% formic acid and 2 mM ammonium formate) and mobile phase B (acetonitrile with 0.1% formic acid and 2 mM ammonium formate) at a flow rate of 0.4 mL/min. Table 2 shows the relevant mass spectrometric operating parameters. The conditions were determined using a central composite design (CCD) (Supplementary Table S2; Supplementary Figure S1).

**TABLE 2:** Mass spectrometric operating parameters for LC-MS/MS analysis of the tested drugs and their metabolites (ESI(+) ionization; gas (nitrogen) flow: 6.0 L/min, 35 psi, spray voltage: 4000 V).

Drug/metabolite (abbreviation)	Precursor ion m/z [M + H]+	Product ion m/z Q1	Product ion m/z Q3	Drying gas temperature [°C]	Fragmentor [V]
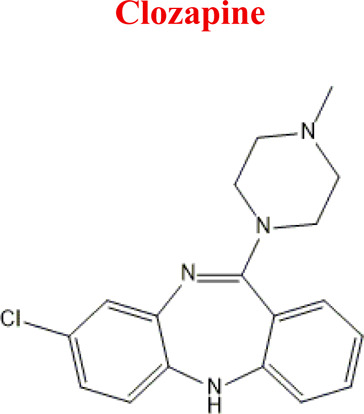	327	270	192	320	70
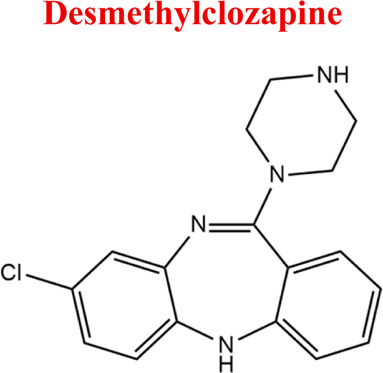	313	192	227	320	70
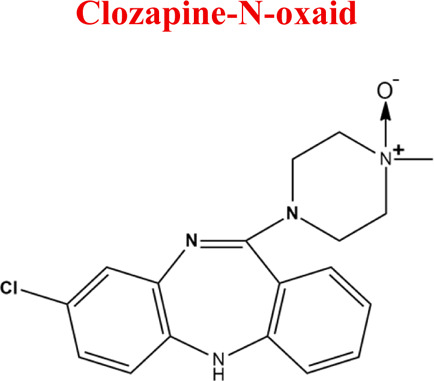	343	192	256	320	70
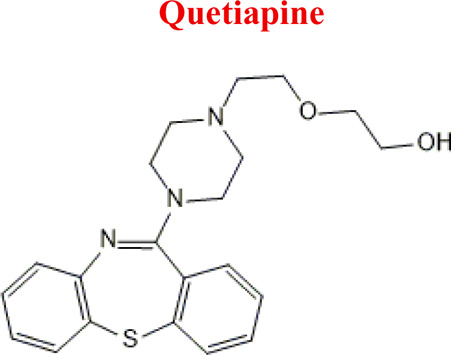	384	253	221	320	150
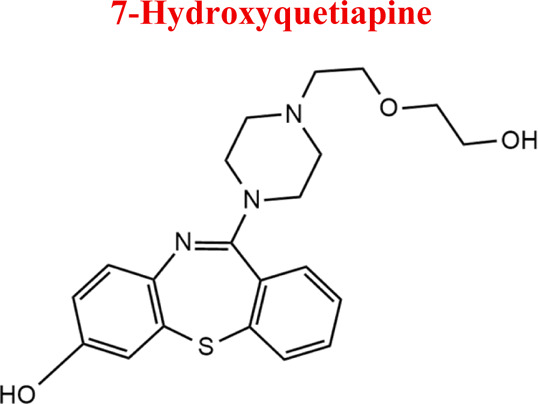	400	269	237	320	150
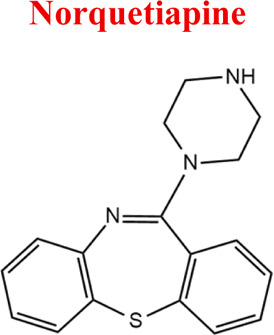	296	210	183	320	150
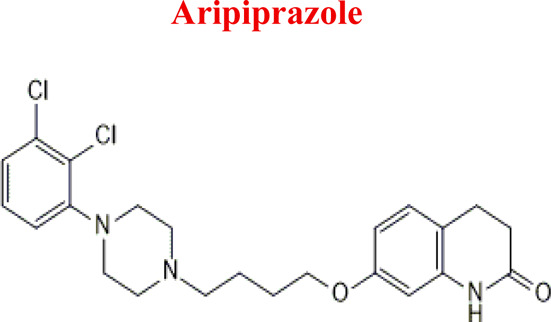	448	287	176	290	70
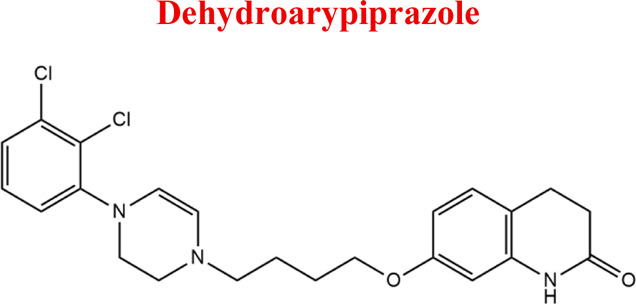	446	285	188	290	70
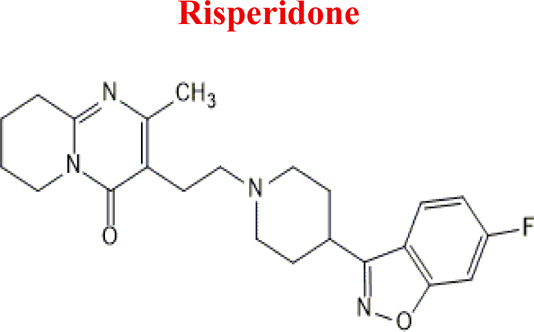	411	191	215	320	110
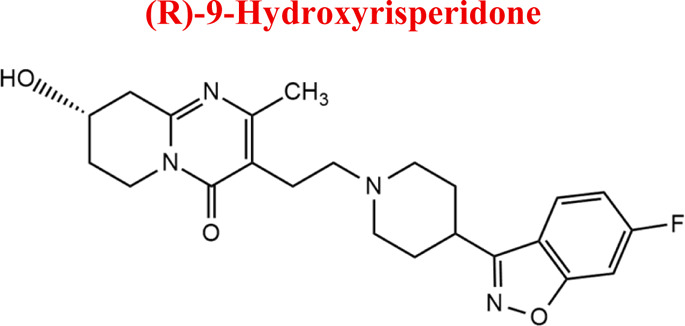	427	207	---	320	110
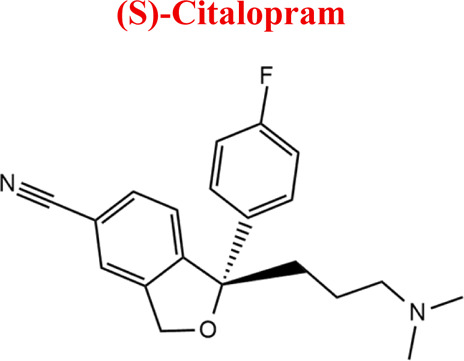	325	109	234	350	110
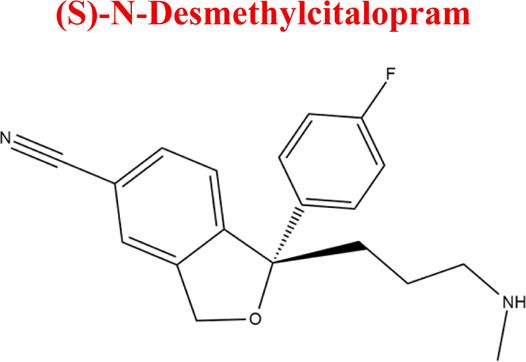	311	109	---	350	110
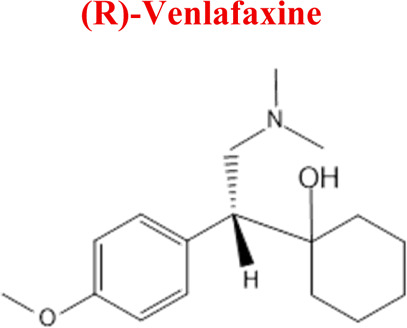	278	260	121	290	110
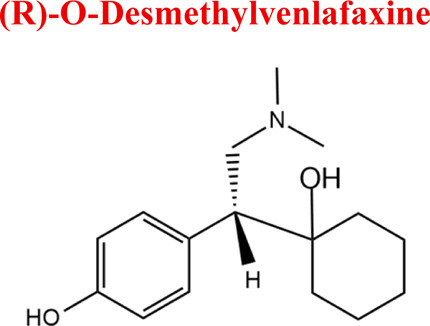	264	246	107	290	110
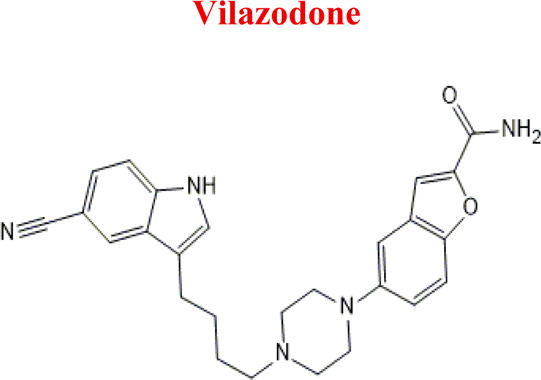	442	155	---	350	150
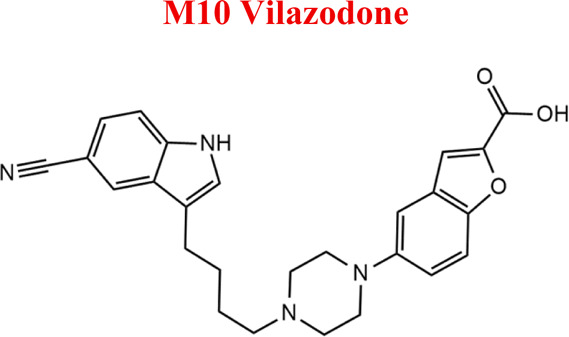	443	143	284	350	150
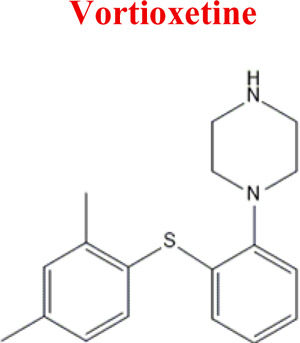	299	203	163	320	110
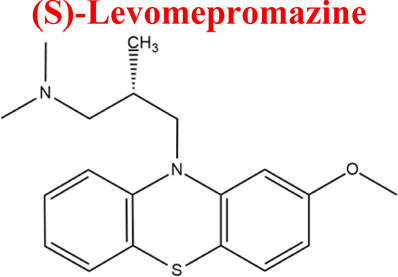	329	100	210	320	110
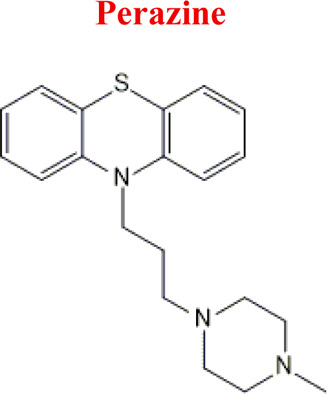	340	141	209	320	110
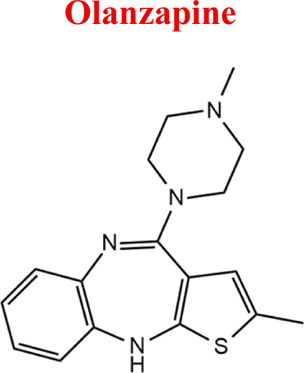	312	256	198	350	110
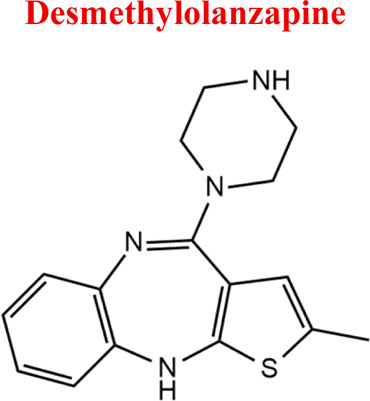	299	256	213	350	110

### 2.5 Biological sample preparation

Whole blood samples (peripheral venous blood) were collected from patients. Sample collection was performed by doctors of the II Department of Psychiatry of the Ludwik Rydygier Provincial Polyclinical Hospital in Torun. The study was approved by the Nicolaus Copernicus University Bioethics Committee in Torun (decision no. 477/2021). All study participants were informed verbally and in writing about the purpose of the study and completed a questionnaire and a declaration of voluntary consent to participate in the study. Raw blood samples (2 mL) were collected from adult patients treated with selected psychotropic drugs (aripiprazole, clozapine, quetiapine, venlafaxine, vortioxetine) after oral administration of the drug (minimum period of 2 weeks). A chiral drugs in a pure enantiomeric form were applied. It was significant because enantiomers can have different biological effects, therapeutically beneficial, potentially harmful, or inactive. It was crucial for optimizing therapeutic efficacy, minimizing adverse effects, and advancing drug development and personalized medicine. The pharmacokinetics of antidepressants is stereoselective and predominantly favors one enantiomer. The use of pure enantiomers offers (i) better specificity than the racemates in terms of certain pharmacological actions, (ii) enhanced clinical indications, and (iii) optimized pharmacokinetics. Therefore, controlling the stereoselectivity in the pharmacokinetics of antidepressive drugs is of critical importance in dealing with depression and psychiatric conditions. Whole blood samples were collected in tubes. Then, after centrifugation of the morphotic elements, the plasma was frozen (−80 °C) and thawed only immediately before performing the analytical procedure to evaluate *in vivo* metabolism.

### 2.6 Biological samples - isolation and enrichment of selected components by using microextraction on packed sorbent (MEPS)

MEPS was used to reach this objective.

Plasma-fortified samples were used to select the extraction parameters. A summary of the tested MEPS sorbents is shown in Supplementary Table S3.

### 2.7 Drug incubations in the microsomal fraction of human liver cells

*In vitro* drug incubations were carried out in the microsomal fraction of liver cells to obtain and analyze metabolites. Supplementary Table S4, shows the composition of the microsomal fractions used in this study and their activity. The drug incubation (10 μL of a 2 mM solution of the respective drug in the phosphate saline buffer) with human liver microsomes (CYP450; 10 μL of 20 mg/mL) was performed in 35 μL phosphate buffer (BR) with the addition of 10 μL NADPH (20 mM). All the samples were incubated at 37 °C for 0, 15, 30, 60, 90 or 120 min and shaken during the incubation. The reaction was terminated by the immersion of the samples in an ice bath and the addition of 65 μL ice-cold ACN. After that, all samples were centrifuged for 5–15 min at 5000rpm. Then, the supernatant was analyzed by LC- MS/MS.

### 2.8 Method validation

The method was validated according to the ICH guidelines. The following validation characteristics were addressed: linearity, detection and quantitation limits, accuracy, precision, robustness, selectivity and stability (Supplementary Tables S5–S7).

## 3 Results

### 3.1 Optimization of experimental conditions of EC/MS

The influence of the electrode type, pH, and type of buffer on the EC analysis of psychotropic drugs was discussed, and the most optimal conditions were determined.

**FIGURE 2:**
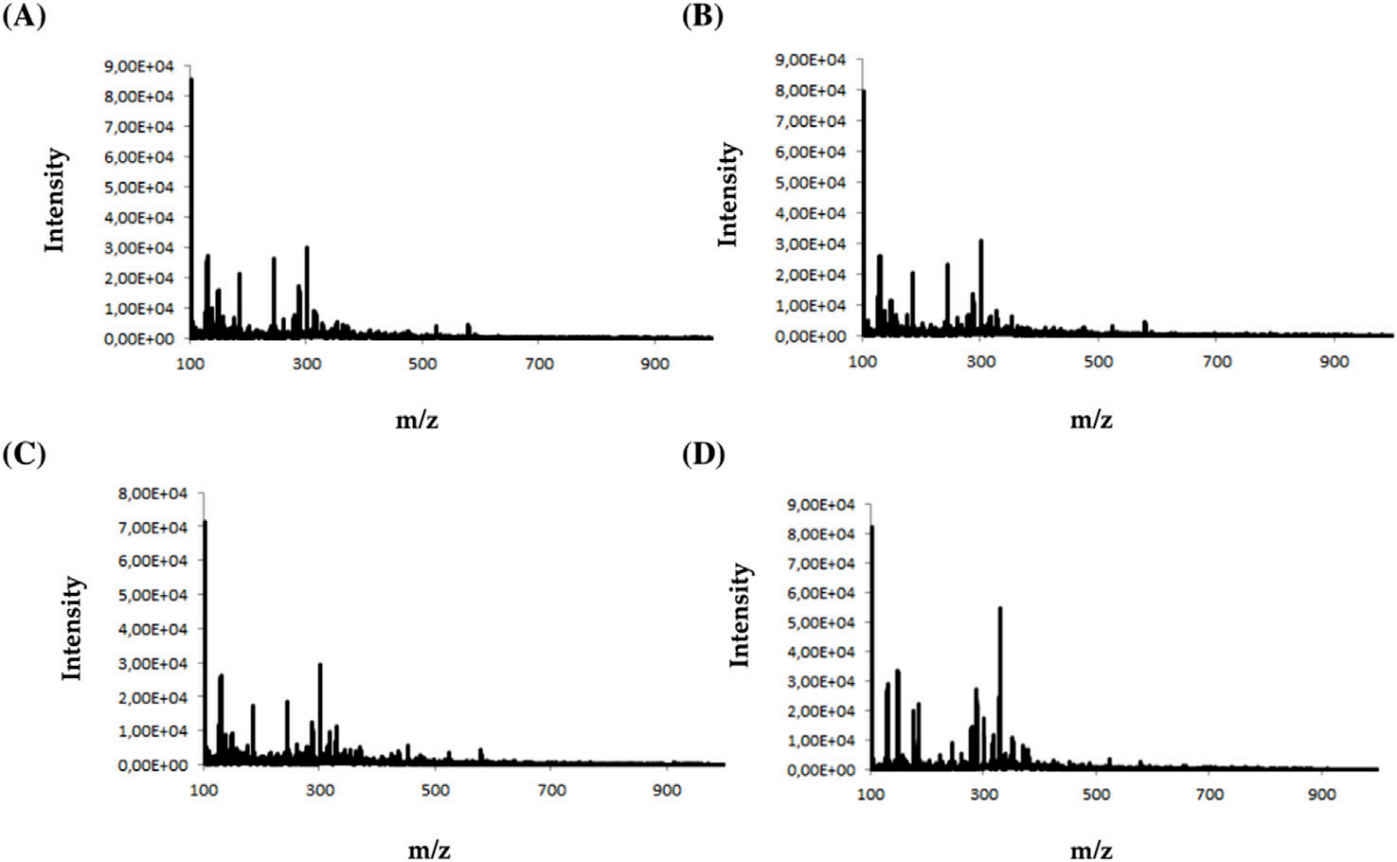
Mass spectra obtained for olanzapine using **(A)** platinum Pt, **(B)** gold Au, **(C)** glassy carbon GC, and **(D)** boron-doped diamond MD electrodes.

During the analysis, four types of electrodes were tested: platinum (Pt), gold (Au), glassy carbon (GC), and boron-doped diamond electrode (MD) consisting of an ultra-thin crystalline diamond layer deposited on top of a silicon substrate. The criterion for choosing a working electrode was the amount and intensity of the signals on the mass spectrum. Figure 2 shows an example of MS spectra obtained for olanzapine using four different working electrodes.

Analyzing the obtained MS spectra of the tested compounds, it can be concluded that the highest number of signals is possible when using a boron-doped diamond MD electrode. The efficiency of this working electrode is probably related to the possibility of conducting electrochemical processes in a wider potential range (0–3000 mV).

**FIGURE 3:**
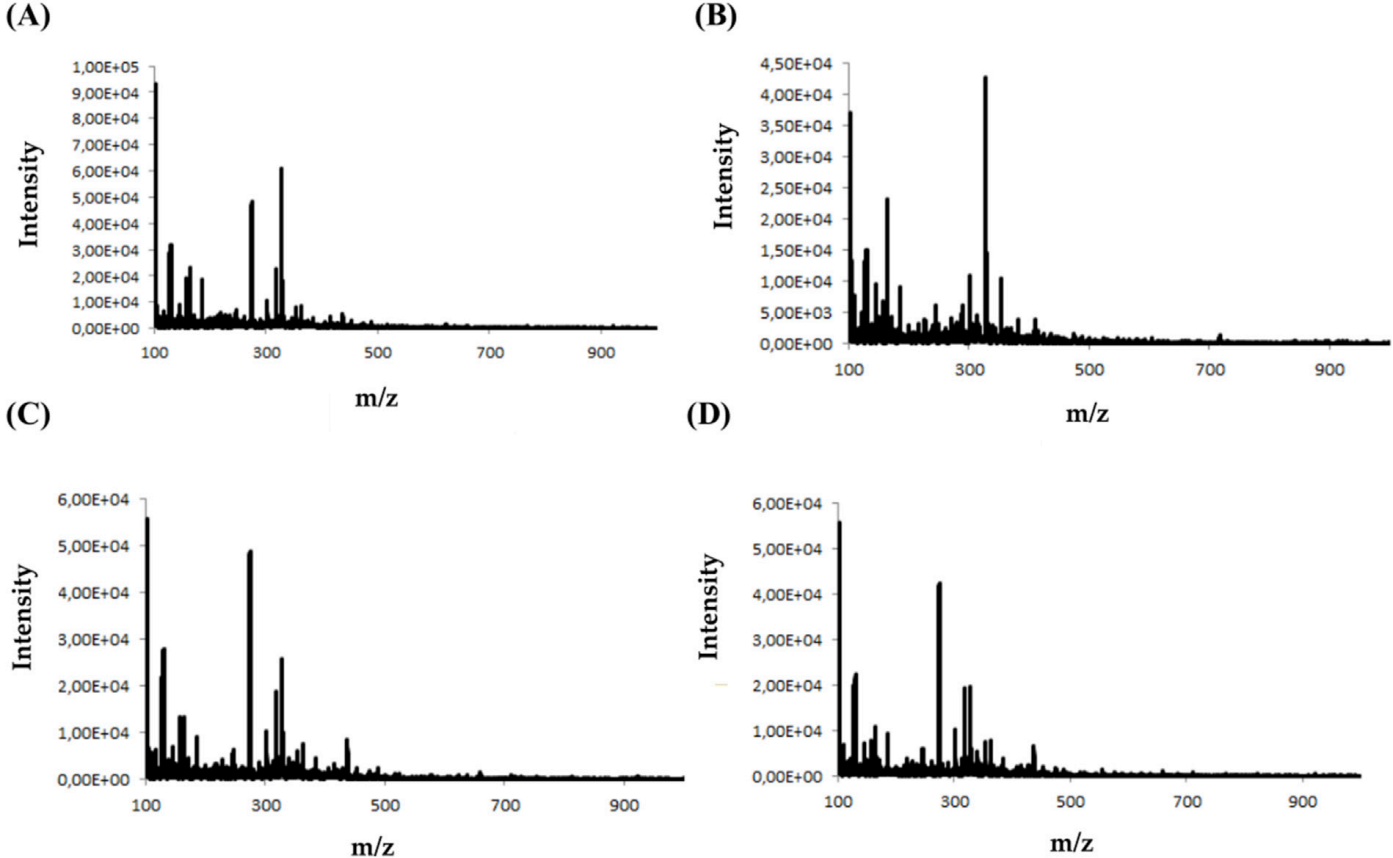
Mass spectra obtained for clonazepam in ammonium formate buffer at pH 3 **(A)** ammonium acetate at pH 7**(B)** ammonium acetate at pH 5 **(C)** and ammonium acetate at pH 9 **(D)**.

During the analysis, four types of buffer (concentration 10 mM) with different pH were tested: ammonium formate buffer at pH 3 (A), ammonium acetate at pH 7(B), ammonium acetate at pH 5 (C), and ammonium acetate at pH 9 (D). The criterion for selecting the appropriate buffer and pH was the amount and intensity of signals in the mass spectra for selected psychotropic drugs. Figure 3 shows an example of MS spectra obtained for clonazepam in four different MD electrode solutions.

Analyzing the mass spectra of the studied compounds (Figure 3), it can be concluded that the highest number and intensity of signals correspond to electrochemical processes using the mobile phase at pH values of 5 and 7. The ammonium acetate buffer was selected for further study due to the highest intensity of signals.

### 3.2 Identification of electrochemical products of psychotropic drugs

EC-MS data was collected for the eleven psychotropic drugs studied in this work. Psychotropic drug intensity decreases with the increasing potential. This suggests a degradation of the parent compound. At the same time, an increase in signal intensity is observed for individual electrochemical transformation products.

**FIGURE 4:**
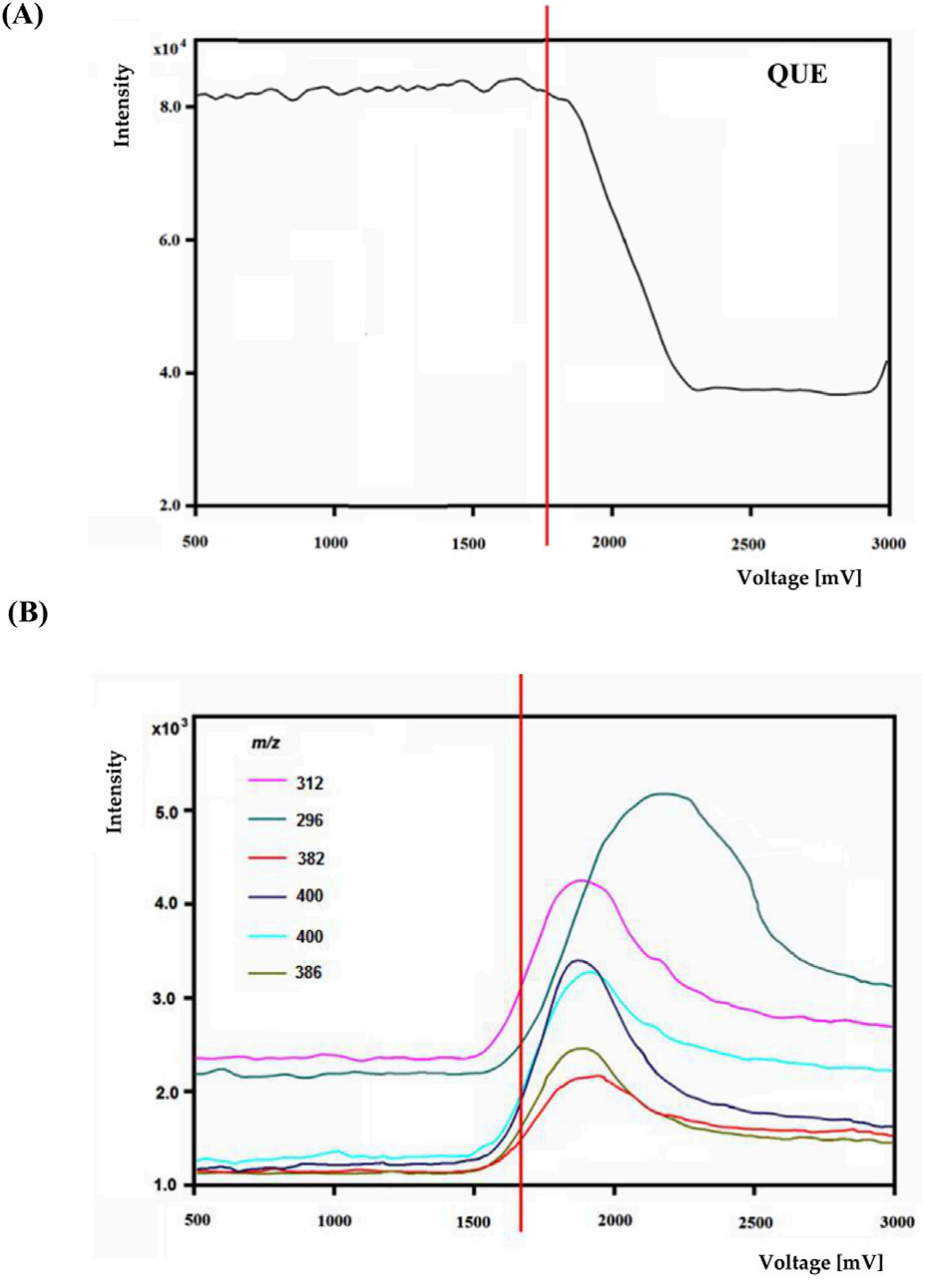
The voltage-mass intensity curves of quetiapine **(A)** and selected oxidation product ions **(B)**.

An exemplary mass voltammogram recorded on a BDD electrode in a potential range from 0 to 3000 mV is shown in Figure 4 for quetiapine.

The most efficient formation of detectable electrochemical transformation products was observed in a potential range from 1800 to 2,200 mV. At higher potentials, the intensity electrochemical transformation products decreases. Similar effects occurred for other psychotropic drugs. Based on potential-dependent signal intensities and MS/MS spectra, structures were proposed for electrochemical transformation products (see Table 3).

**TABLE 3:** The proposed structures of electrochemical oxidation products of target compounds.

*m/z*	Intensity	Proposed metabolic reaction	Proposed Chemical Structural
Quetiapine [M+H]^+^ =384			
296	Major	*N*-dealkylation	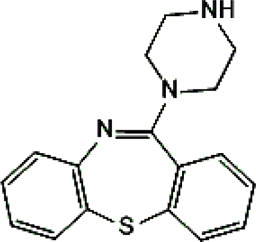
312	Major	*N*-dealkylation and hydroxylation	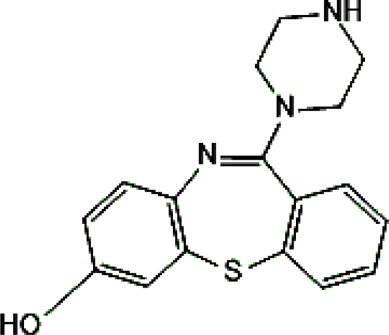
401	Major	Hydroxylation	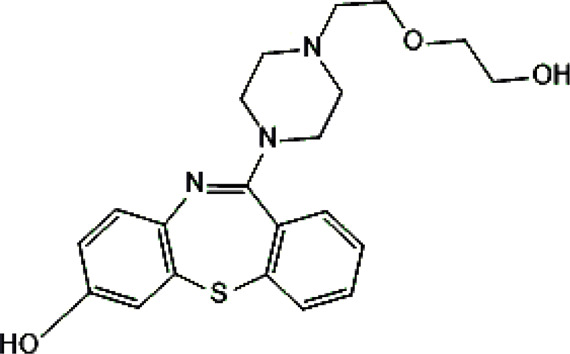
400	Medium	*S*-Oxidation	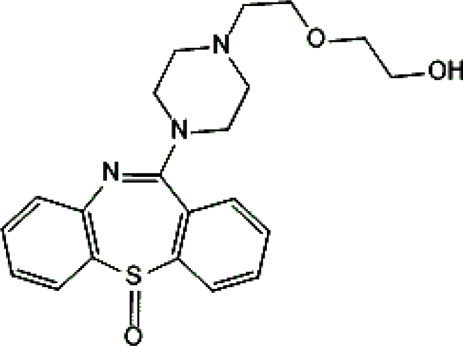
689	phase II	GSH conjugation	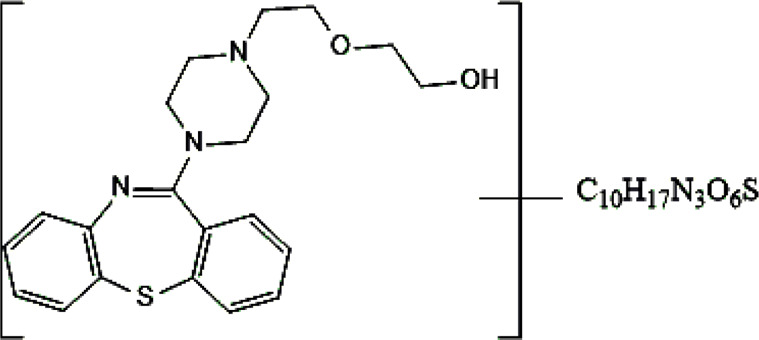
386	Minor	Hydrogenation	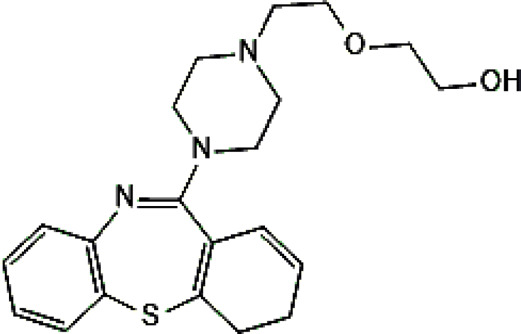
382	Minor	Dehydrogenation	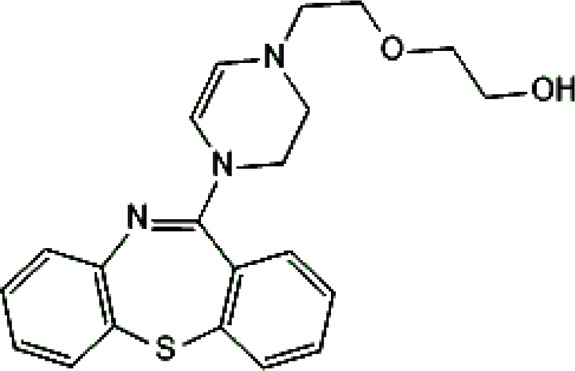
Levomepromazine [M+H]^+^= 329			
315	Major	Demethylation	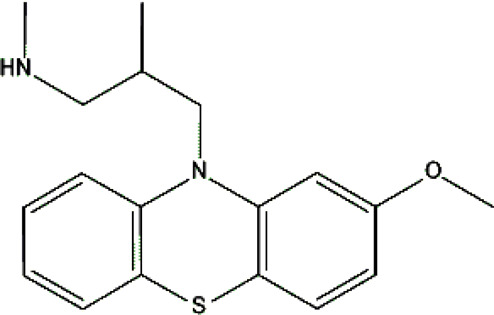
345	Major	S-Oxidation	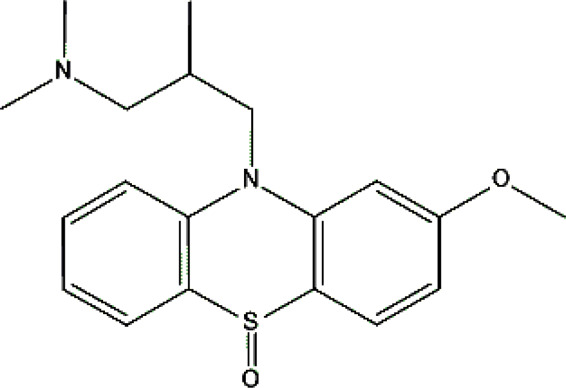
327	Minor	Oxidation	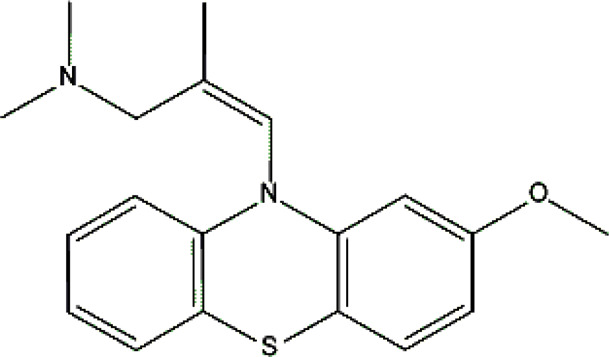
331	Minor	Reduction	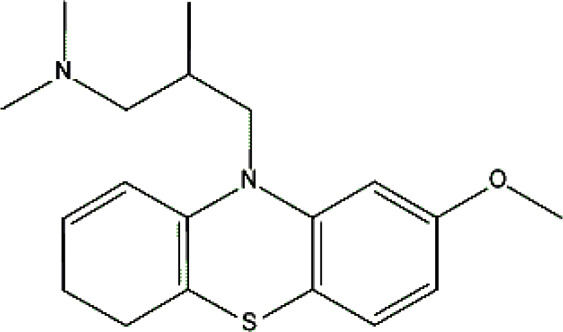
Olanzapine [M+H]^+^= 312			
299	Major	Demethylation	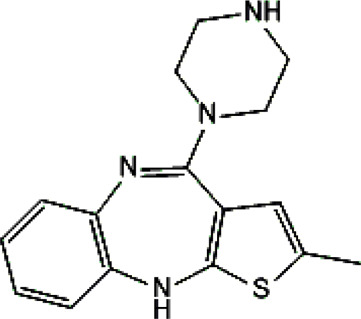
329	Major	Hydroxylation	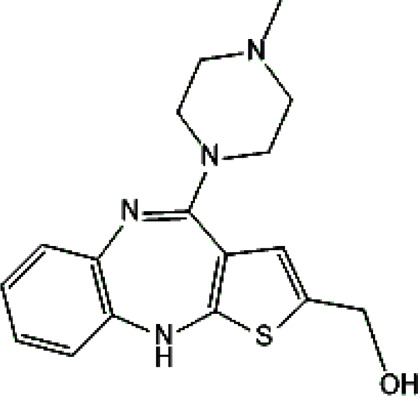
329	Medium	S-Oxidation	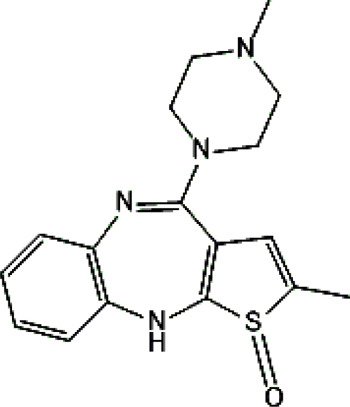
Perazine [M+H]^+^= 340			
326	Major	Demethylation	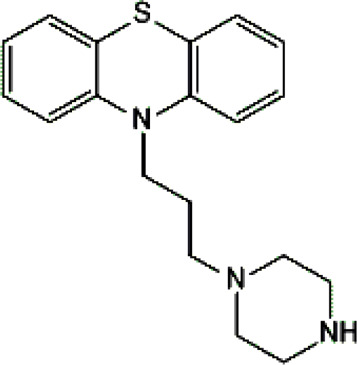
356	Medium	Hydroxylation	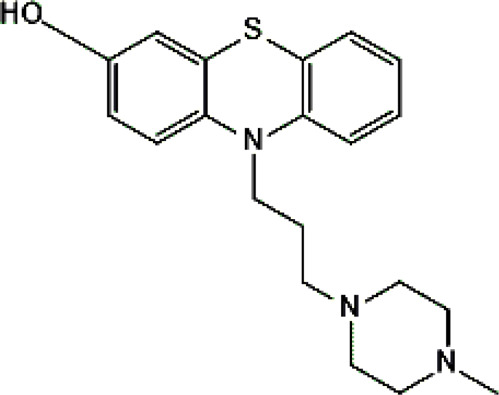
338	Minor	Oxidation	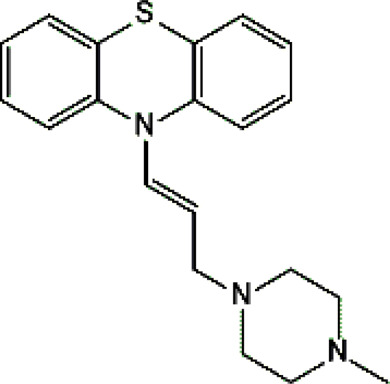
342	Minor	Reduction	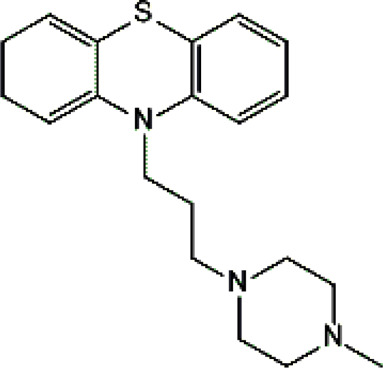
Clozapine [M+H]^+^= 327			
313	Major	Demethylation	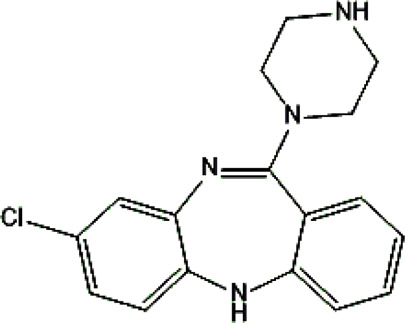
343	Major	Oxidation	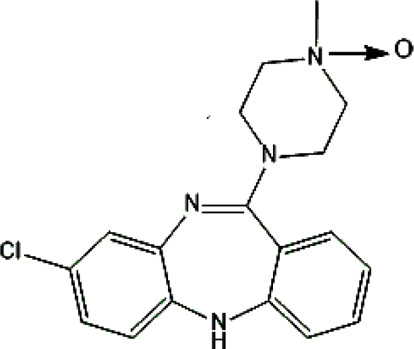
632	phase II	GSH conjugation	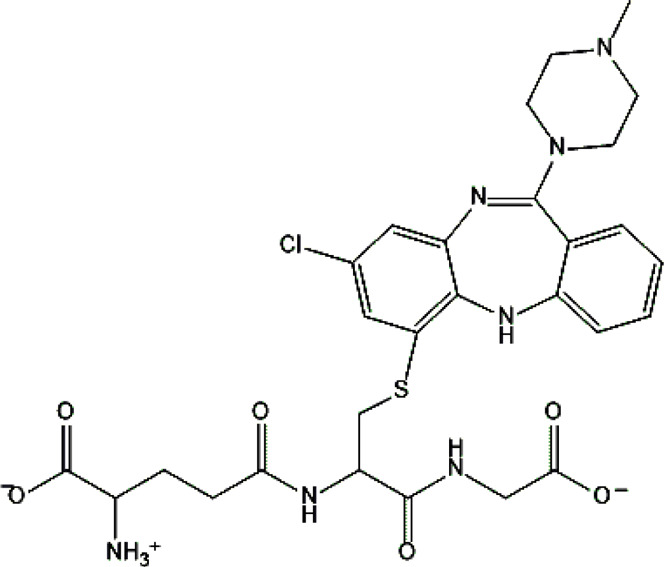
325	Medium	Oxidation	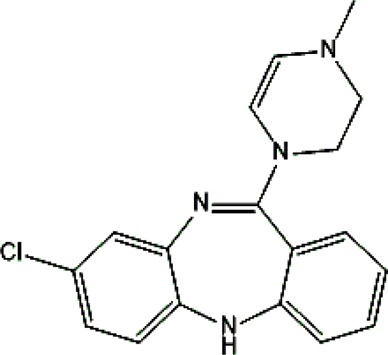
329	Minor	Reduction	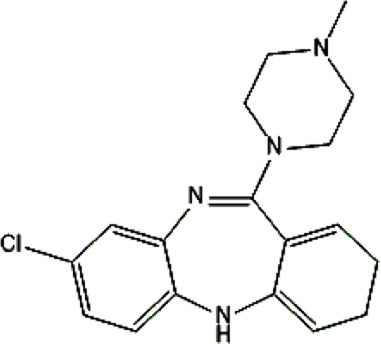

The structural formulas presented in the article are exemplary structures of the obtained and identified electrochemical products. A nuclear magnetic resonance (NMR) spectroscopic analysis should be performed to determine the exact structures of the obtained products. The path of the proposed electrochemical reactions including reactions characteristic of the first and second phases of biotransformation are shown in Supplementary Figures S3–S7. The chemical structures were proposed based on EC-MS/MS experiments and information available in literature. Four proposed electrochemical products were identified for the electrochemical simulation of vortioxetine (Supplementary Figure S3). The electrochemical product M1 observed for *m/z* = 315 indicates a hydroxylation reaction probably at the methylene group site; M2 for *m/z* = 329 is a continuation of the response for M1 and the formation of a carboxyl group at the methylene group site; M3 and M4 are the typical attachment and detachment of two hydrogen atoms that are present in virtually every electrochemical simulation. Four proposed electrochemical products were identified for the electrochemical simulation of aripiprazole (Supplementary Figure S4). The electrochemical product M1 and M4 are typical attachment and detachment reactions of two hydrogen atoms (*m/z* = 446 and *m/z* = 450). In contrast, M2 characteristic for *m/z* = 464, is a hydroxylation reaction of the aromatic ring; M3 for *m/z* = 432 is the result of detachment of an oxygen atom from the cyclic ring. Five proposed electrochemical products were identified for the electrochemical simulation of citalopram (Supplementary Figure S5). The electrochemical product M1 is probably an additional reaction of an oxygen atom on a nitrogen atom; M2 for *m/z* = 311 indicates a demethylation reaction at the nitrogen atom. Attaching an oxygen atom to a nitrogen atom, M3 and M4 are the typical attachment and detachment of two hydrogen atoms (*m/z* = 327 and *m/z* = 323)—the product M5 for *m/z* = 297 results from two demethylation reactions at the nitrogen atom. Five proposed electrochemical products were identified for the electrochemical simulation of venlafaxine (Supplementary Figure S6). The electrochemical product M1 for *m/z* = 262 is probably a dehydroxylation reaction at the cyclic ring site; M2 and M4 are typical attachment and detachment reactions of two hydrogen atoms (*m/z* = 280 and *m/z* = 276); M3 for *m/z* = 294 is probably an oxygen atom attachment reaction at the nitrogen atom. The product M5 for *m/z* = 264 results from demethylation at the oxygen atom. Eight proposed electrochemical products were identified for the electrochemical simulation of vilazodone (Supplementary Figure S7). Electrochemical products M1, M3, and M5-7 are hydroxylation reactions of the attachment of one or more OH groups at different sites of vilazodone. Product M2 is the result of dehydrogenation, while M4 is probably the attachment of two oxygen atoms to the cyclic ring of the piperazine derivative. The *m/z* = 747 ion for the electrochemical product M8 indicates the addition of glutathione to vilazodone.

### 3.3 *In silico* prediction of metabolism

*In silico* prediction of metabolism was performed on GloryX and Biotransformer3.0 free ware. Moreover, a robust approach to ranking the predicted metabolites is attained by using the SoM (sites of the metabolism) probabilities predicted by the FAME 3 (Supplementary Table S8) machine learning models to score the predicted metabolites. Predicted derivatives with an assigned score were considered probable or minority and less likely. Overall, Phase I and Phase II metabolites were predicted. Major Phase I metabolites included hydroxylated and oxidated derivatives. Major Phase II metabolites included glutathione conjugated, sulfo conjugated, and methylated derivatives.

### 3.4 HLMs–*in vitro* incubation

Incubating the microsomal fraction of liver cells without NADPH at 37 °C for a specified time did not affect the amount of the compound in the reaction mixture. The results indicate that the enzymatic assays should be carried out with NADPH cofactor. The degree of conversion of psychotropic drugs depended on the concentration and incubation time of NADPH—the results of the experiments illustrated for clozapine are shown in Supplementary Figure S8. In the graph, the substrate concentration decreased linearly with the progress of the reaction. No signals from other substances were observed during the analyses when the drug was incubated without the cofactor. The results were also confirmed for the other psychotropic medications.

In the study, we determined the chemical structures of the resulting metabolic biotransformation products of the studied drugs. Mass spectra were recorded for each analyte tested (substrates) and potential metabolites (products) using the HPLC-ESI/MS technique. The Mass Hunter Metabolite ID computer program was helpful during the analysis of the results. This analytical tool compared the analyte signals with the control samples by comparing their retention times, MS spectra, and area fields. MS spectra and [M + H]^+^ fragmentation spectra for the proposed metabolites were recorded for the available metabolite standards of selected psychotropic drugs. This was not possible for the other identified potential metabolites due to the lack of commercially available standards or their high price. All the commercially available standards of metabolites discussed in the manuscript are the predominant metabolites for each drug (pharmacologically active metabolites).

**FIGURE 5:**
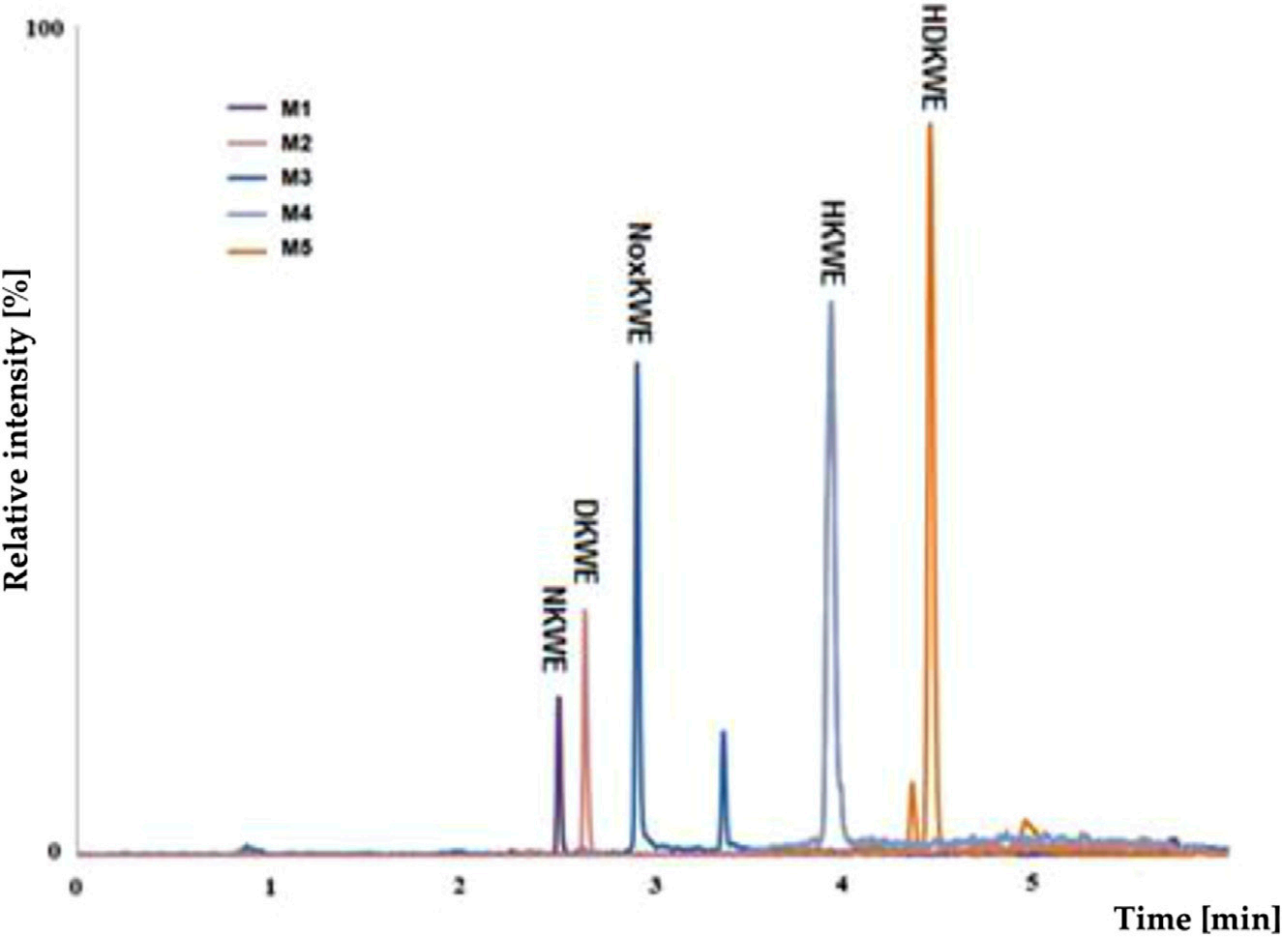
MS chromatogram for the quetiapine metabolites (M1-M5).

Using pre-optimized mass spectrometer analysis conditions, quetiapine (QUE as a model compound) was identified as [M + H]+ for *m/z* = 384. Fragmentation of QUE and other drugs was performed to obtain data to interpret MS spectra for potential metabolites of selected drugs. Incubation on the example QUE along with liver enzymes of the microsomal fraction in the presence of NADPH as a cofactor for enzymatic reactions led to the formation of five potential metabolites (S/N˃5), (Figure 5).

The analysis of the MS chromatogram below indicates that five (M1-M5) metabolites of phase I of the biotransformation reaction were formed. The potential metabolites were numbered M1-M5 based on their retention times. The chemical structures proposed for them were confirmed through parallel detection of 2 MS/MS fragmentation transitions for the selected molecule (potential metabolite).

Using a mass spectrometer, the various potential metabolites were identified and confirmed based on the proposed fragmentation mechanism (Supplementary Figure S9; Supplementary Figure S10). The specific fragments were identified on the characteristic ions, indicating the relevant functional groups in the molecule. Mass ions with characteristic m/z values were observed on MS spectra of the potential metabolites of microsomal fraction enzymes (Table 4). After incubation, the potential metabolites were formed due to the phase I biotransformation. Table 4 characterizes the retention times, chemical structures, m/z ratio, elemental composition, and a proposed biochemical reaction depending on the selected psychotropic drug. Interpreting the collected mass spectra (MS) of the peaks of the obtained metabolites allows us to conclude that the other studied psychotropic drugs are susceptible to enzymatic transformations in the presence of selected cosubstrates.

**TABLE 4:** Retention time, characteristic masses, and elemental composition for *m/z* [M + H]^+^ potential metabolites.

Psychotropic drug*	Potential metabolites	Retention time (min)	*m/z* [M + H]^+^	Chemical formula	Proposed metabolic reaction
*Quetiapine*	*M1 QUE*	2,49	296	C_17_H_17_N_3_S	N-dealkylation
	*M2 QUE*	2,63	340	C_19_H_21_N_3_OS	O- dealkylation
	*M3 QUE*	2,91	400	C_21_H_25_N_3_O_3_S	Reduction
	*M4 QUE*	3,98	400	C_21_H_25_N_3_O_3_S	Hydroxylation
	*M5 QUE*	4,46	312	C_17_H_17_N_3_OS	N-dealkylation, Hydroxylation
*Aripiprazole*	*M1 DARP*	7,15	446	C_23_H_25_Cl_2_N_3_O_2_	Oxidation
	*M2* *DCPP*	6,23	232	C_10_H_11_Cl_2_N_2_	N-dealkylation
	*M3* *DCPQ*	5,47	250	C_13_H_15_NO_4_	N-dealkylation
*Clozapine*	*M1* *NoxKLO*	6,87	343	C_18_H_19_ClN_4_O	Reduction
	*M2* *DKLO*	6,12	313	C_17_H_17_ClN_4_	N-Demethylation
*Venlafaxine*	*M1* *DWEN*	5,67	264	C_16_H_25_NO_2_	O- Demethylation
	*M2* *NoxWEN*	4,94	294	C_17_H_27_NO_3_	Reduction
*Vortioxetine*	*M1* *M8*	4,58	315	C_18_H_22_N_2_SO	Hydroxylation
	*M2* *M0*	4,25	329	C_18_H_22_N_2_SO_2_	Oxidation of the methyl group to carboxylic acid

Psychotropic drug* that used to be involved in enzymatic reaction.

Taking everything into consideration human microsomes are a suitable model for studying and predicting the metabolic transformations of the analytes that may occur in the patient’s body. All proposed phase I reactions of metabolism are typical of processes catalyzed by the isoenzymes of the cytochrome P450 group.

### 3.5 MEPS–real samples preparation

The isolation of the tested compounds was carried out using different MEPS sorbents and the eVol^®^ automatic electronic syringe. Depending on the filling used, different efficiency, selectivity, and reproducibility of extraction concerning selected psychotropic drugs and their metabolites were obtained.

Analyzing the obtained results, it can be concluded that the use of the C18 sorption for the isolation of selected analytes from contaminated plasma samples allowed for higher recoveries (average recovery 94.35% ± 1.72%) than in the case of the use of other sorption beds, while maintaining reasonable repeatability determinations (average CV value was approx. 3%). An additional advantage was the possibility of using the C18 sorption bed in a wide pH range (3–9) of the tested samples. A comparison of the obtained recoveries using selected MEPS sorption beds is presented in Supplementary Table S9.

Sorbents with octadecylsilane filling showed high extraction efficiency (recovery) and repeatability of the tested drugs. In contrast, the remaining sorption beds tested in the study had low extraction efficiency for most tested compounds. In addition, silica gel, modified with octadecyl groups (C18), is the most commonly used sorption material. Although the C18 sorbent is non-polar, it can be used for both polar and non-polar compounds. This is due to their long octadecyl chain, in which drugs, as non-polar compounds, get stuck and easily wash out with a polar solvent.

The next step was the selection of the solvent used for the elution of analytes from sorption. Depending on the type of eluting solvent used, various recoveries of analytes from plasma samples were obtained and related to their diversified polarity. In the case of methanol, the most minor average recovery of psychotropic drugs was obtained, which was about 50%. Modifying the solvent composition by adding water resulted in a significant increase in the average recovery of analytes to about 75%. Elution with buffer solutions at pH = 3, 5, and 9 did not improve extraction efficiency compared to methanol: water (50:50; *v/v*). The best results (mean recovery 98.16% ± 1.75%) were obtained using a mixture of acetonitrile: methanol: water (5:3:2; *v/v/v*) as the extraction medium. Supplementary Table S9, summarizes the results obtained after using various sorbents for extraction and an example solvent for the elution of analytes (acetonitrile: methanol: water (5:3:2; *v/v/v*)). The next step in selecting appropriate extraction conditions was to study the effect of the number of extraction cycles (1, 2, or 4). The best recovery for each analyte was obtained by performing two extraction cycles, where recoveries ranged between 91% and 99%.

### 3.6 Analysis results of biological samples from patients

**FIGURE 6:**
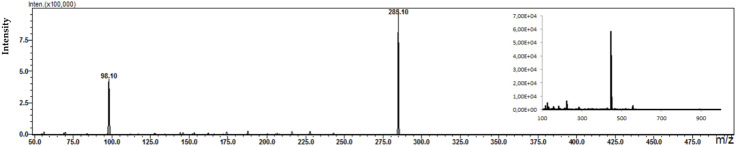
Fragmentation spectrum with MS spectrum for the identified metabolite (DARI) in the plasma sample of a patient treated with ARI (30 mg dose).

**FIGURE 7:**
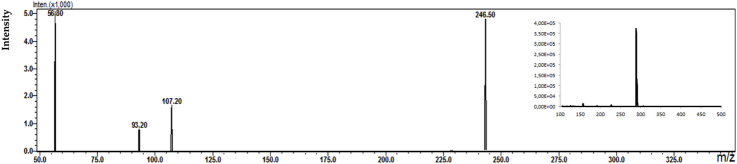
Fragmentation spectrum with MS spectrum for the identified metabolite (DWEN) in a plasma sample of a patient treated with WEN (225 mg dose).

**FIGURE 8:**
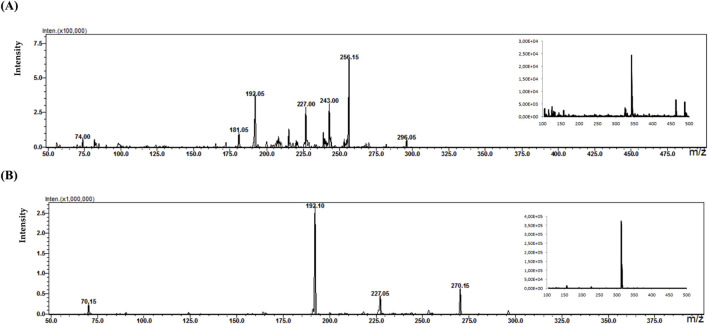
Fragmentation spectra with MS spectra for the identified metabolites [NoxKLO **(A)** and DKLO **(B)**] in a plasma sample of a patient treated with KLO (150 mg dose).

**FIGURE 9:**
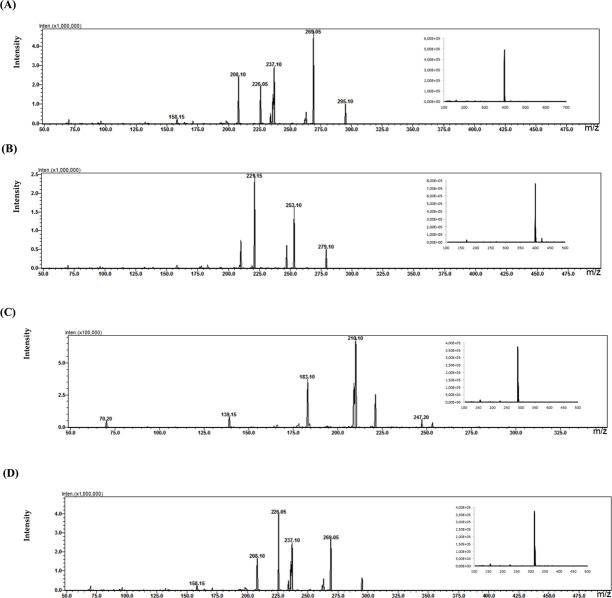
Fragmentation spectra with MS spectra for the identified metabolites [HQUE **(A)** SoxQUE **(B)** NQUE **(C)** and HDQUE **(D)**] in a plasma sample from a patient treated with QUE (750 mg dose).

In the study of biological samples, the microextraction technique on packed sorbent (MEPS) was used to isolate and enrich analytes from plasma samples. Each patient was treated with a different psychotropic substance. The identified metabolite in the plasma sample of a patient treated with aripiprazole (30 mg dose) was its primary metabolite, dehydroarypiprazole (DARI). The molecular ion of this compound is characteristic of *m/z* = 446. The MS fragmentation spectrum for DARI with a distinctive signal for *m/z* = 285 (with the highest intensity) is confirmed by the MS spectrum for the sample of the metabolite standard (Figure 6). In the case of a plasma sample from a patient treated with venlafaxine (225 mg dose), the MS fragmentation spectrum shows two signals for *m/z* = 246 (with the highest intensity) and *m/z* = 107, which are characteristic ions for its metabolite O-desmethylenlafaxine. The result is consistent with the analyses performed for the standard metabolite of this drug, DVEN (Figure 7). For a plasma sample of a patient treated with clozapine, the MS and MS fragmentation spectra in Figure 8 identified the primary two metabolites: clozapine N-oxide and N-desmethylclozapine. Figure 8A shows the two fragmentation ions *m/z* = 192 and *m/z* = 256 (with the highest intensity) characteristic of NoxCLO. In contrast, Figure 8B shows two fragmentation ions for *m/z* = 192 (with the highest intensity) and *m/z* = 227, characteristic of DCLO. Four of its metabolites were identified in the plasma sample of a patient treated with quetiapine (750 mg dose). Figure 9A shows the MS spectrum for 7-hydroxy quetiapine, as indicated by the molecular ion *m/z* = 400 and the characteristic signals for *m/z* = 269 (highest intensity) and *m/z* = 237 on the MS fragmentation spectrum. The result is consistent with the data obtained for the HQUE metabolite standard. Figure 9B shows a spectrum with a molecular ion for *m/z* = 400 and characteristic fragment ions for SoxQUE *m/z* = 221 (highest intensity), *m/z* = 253 (medium intensity) and *m/z* = 279. Figure 9C shows an MS spectrum for NQUE, as indicated by the presence of a molecular ion for *m/z* = 296 and characteristic fragment ions for *m/z* = 210 (highest intensity) and *m/z* = 183 (medium intensity). Figure 9D shows three fragmentation ions for *m/z* = 269, m*/z* = 237 (with medium intensity), and *m/z* = 226 (with highest intensity) characteristic of HDQUE.

**FIGURE 10:**
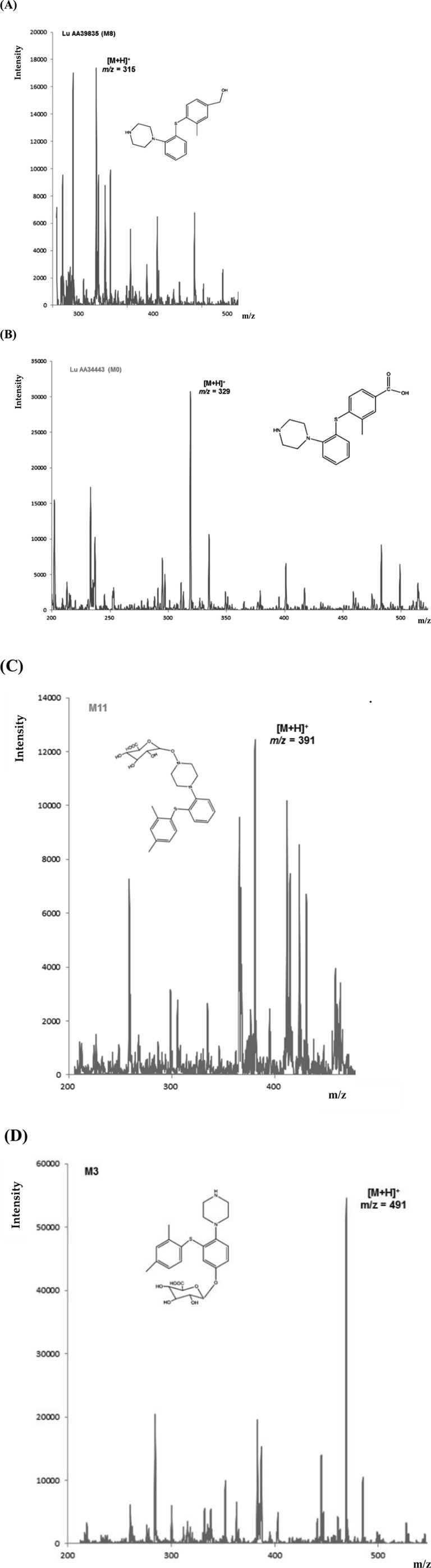
MS spectra for the identified metabolites [M8 **(A)**, M0 **(B)**, M11 **(C)**, and M3 **(D)**] in a plasma sample of a patient treated with VOR (20 mg dose).

For a patient treated with vortioxetine (20 mg dose), four metabolites were identified in a plasma sample. Figure 10 shows MS spectra with signals from the precursor ion of the metabolites: *m/z* = 315 for M8 (A), *m/z* = 329 for M0 (B), *m/z* = 391 for M11 (C), and *m/z* = 491 for M3 (D). These results are confirmed in the literature. Due to the lack of commercially available metabolite standards or their high price, MS fragmentation was not performed.

Based on the results, it can be concluded that the MEPS approach can be successfully adopted at the stage of biological sample preparation as a microextraction technique for isolating drug metabolites.

## 4 Discussion

The results obtained for experiments by electrochemical simulation (EC) and incubation of the test drugs with liver cell microsomal fraction (HLM) enzymes with the results of biological sample analysis for the five selected drugs (QUE, CLO, ARI, VEN, and VOR) were collected in Table 5. Only the identification of their electrochemical products was made using instrumental technique (EC for the remaining drugs). Accordingly, for QUE, some of the identified electrochemical products can be identified with three metabolites, i.e., hydroxy quetiapine, no quetiapine, and quetiapine N-oxide. For CLO, clozapine N-oxide and desmethylozapine, respectively; for ARP, dehydroarypiprazole was formed, while for VEN, desmethyl venlafaxine was identified, and for VOR, two metabolites described in the literature, M8 and M0. In the case of VEN, one of the metabolites (venlafaxine N-oxide) identified when the drug was incubated in the presence of enzymes of the microsomal fraction of liver cells and as an electrochemical product during simulation was not confirmed in the actual patient sample.

**TABLE 5:** Summary of results for electrochemical studies (EC), microsomal incubations (HLM) and biological samples (human plasma).

Psychotropic drug	Identified compound	*m/z* [M + H]^+^	Confirmed by
*QUE*	*HQUE*	400	EC, HLM, PLASMA
	*NQUE*	296	EC, HLM, PLASMA
	*NoxQUE*	400	EC, HLM, PLASMA
	*DQUE*	340	HLM, PLASMA
	*HDQUE*	312	HLM, PLASMA
	*SoxQUE*	400	PLASMA
	*+2H*	386	EC
	*-2H*	382	EC
	*with GSH*	689	EC
*CLO*	*NoxCLO*	343	EC, HLM, PLASMA
	*DCLO*	313	EC, HLM, PLASMA
	*-2H*	325	EC
	*+2H*	329	EC
	*with GSH*	632	EC
*ARI*	*DARI*	446	EC, HLM, PLASMA
	*+2H*	450	EC
	*+O*	464	EC
	*-O*	432	EC
	*DCPP*	232	HLM
	*DCPQ*	250	HLM
*RIS*	*HRIS*	427	EC
	*NoxRIS*	427	
	*-2H*	409	
	*+2H*	413	
	*-O*	395	
	*with GSH*	716	
*CIT*	*DCIT*	311	EC
	*NoxCIT*	341	
	*DiDCIT*	297	
	*-2H*	323	
	*+2H*	327	
*VEN*	*DVEN*	264	EC, HLM, PLASMA
	*NoxVEN*	294	EC, HLM
	*-2H*	276	EC
	*+2H*	280	EC
	*-O*	262	EC
*WIL*	*M10WIL*	443	EC
	*Hydroxy, dehydro*	456	
	*Di hydroxy*	474	
	*Tro hydroxy*	490	
	*Dehydro*	440	
	*Hydroxy*	458	
	*Di oxy*	470	
	*with GSH*	747	
*VOR*	*-2H*	297	EC
	*+2H*	301	EC
	*M8*	315	EC, HLM, PLASMA
	*M0*	329	EC, HLM, PLASMA
	*M11*	391	PLASMA
	*M3*	491	PLASMA
*LEV*	*DLEV*	315	EC
	*HLEV*	345	
	*-2H*	327	
	*+2H*	331	
*PER*	*DPER*	326	EC
	*-2H*	338	
	*+2H*	343	
	*+O*	356	
*OLA*	*DOLA*	299	EC
	*NoxOLA*	329	
	*HOLA*	329	
	*-2H*	311	
	*+2H*	315	

The isoenzymes in CYP450 of the applied liver cell microsomal fraction had a relevant composition. Supplementary Table S4, shows the composition of the microsomal fractions used in this study and their activity. If the same analyses were carried out for other biological samples, e.g., urine or cerebrospinal fluid, one would have to deal with the presence of transferases, for example,. The lack of transferases makes it impossible to compare the identified electrochemical products by simulation of the phase II biotransformation resulting from the reaction with glutathione (GSH).

The results demonstrate that the combination of electrochemistry and mass spectrometry is an effective method for simulating drug metabolism. Further development of this technique will probably also allow it to be used as an alternative *in vitro* method for studying oxidation-reduction reactions.

Numerous attempts wee made in the scientific literature to mimic the metabolic reactions of the selected compounds occurring in liver microsomes using chemical systems that do not rely on enzymes. The obtained results and applied approaches are in good accordance with the literature and each other since the same phase I potential metabolites (the most abundant) occurred in all applied model systems. In the case of quetiapine, the sulfoxidation and oxidation are the primary metabolic pathways of this drug. According to *in vitro* studies, cytochrome P450 3A4 metabolizes quetiapine to an inactive sulfoxide metabolite and metabolizes its active metabolite, N-desalkyl quetiapine. CYP2D6 also regulates the metabolism of quetiapine. Three metabolites of N-desalkylquetiapine were identified. Two of the metabolites were identified as N-desalkylquetiapine sulfoxide and 7-hydroxy-N-desalkylquetiapine. CYP2D6 is responsible for metabolizing quetiapine to 7-hydroxy-N-desalkylquetiapine, a pharmacologically active metabolite (Bakken et al., 2012). Clozapine is a substrate for many cytochrome P450 isozymes, particularly CYP1A2, CYP2D6, and CYP3A4. The unmethylated, hydroxylated, and N-oxide derivatives are urine and fecal components. Pharmacological testing has shown the desmethyl metabolite (norclozapine) to have only limited activity, while the hydroxylated and N-oxide derivatives were inactive (Lane et al., 1999). Aripiprazole is metabolized primarily by three biotransformation pathways: dehydrogenation, hydroxylation, and N-dealkylation. Based on *in vitro* studies, CYP3A4 and CYP2D6 enzymes are responsible for the dehydrogenation and hydroxylation of aripiprazole, and CYP3A4 catalyzes N-dealkylation (Kubo et al., 2007). In the case of risperidone the extensively metabolized by hepatic cytochrome P450 2D6 isozyme to 9-hydroxyrisperidone. Risperidone also undergoes N-dealkylation to a lesser extent (Mannens et al., 1994). Citalopram is metabolized mainly in the liver via N-demethylation to its primary metabolite, demethylcitalopram, by CYP2C19 and CYP3A4. Other metabolites include didemethylcitalopram via CYP2D6 metabolism, citalopram N-oxide, and propionic acid derivative (Bezchlibnyk-Butler et al., 2000). Venlafaxine undergoes CYP2D6-mediated demethylation to form its active metabolite O-desmethylvenlafaxine (ODV). Venlafaxine can also undergo N-demethylation mediated by CYP2C9, CYP2C19, and CYP3A4 to form N-desmethylvenlafaxine (NDV), but this is a minor metabolic pathway. ODV and NDV are further metabolized by CYP2C19, CYP2D6, and/or CYP3A4 to form N, O-desmethylvenlafaxine (NODV), and NODV can be further metabolized to form N, N, O-tridesmethylvenlafaxine, followed by a possible glucuronidation (Fogelman et al., 1999). Vilazodone is mainly metabolized by cytochrome P450(CYP)3A4 and to a minor extent by CYP2C19 and CYP2D6 (Chavan et al., 2017). Vortioxetine is extensively metabolized primarily through oxidation via cytochrome P450 isozymes CYP2D6, CYP3A4/5, CYP2C19, CYP2C9, CYP2A6, CYP2C8, and CYP2B6 and subsequent glucuronic acid conjugation. CYP2D6 is the primary enzyme catalyzing the metabolism of vortioxetine to its major, pharmacologically inactive, carboxylic acid metabolite, and poor metabolizers of CYP2D6 have approximately twice the vortioxetine plasma concentration of extensive metabolizers (Kang and Lee, 2009). Levomepromazine is metabolized in the liver and degraded to a sulfoxid-, a glucuronid- and a demethyl-moiety (Kang and Lee, 2009). In the case of olanzapine from the CYP system, the main metabolic enzymes are CYP1A2 and CYP2D6. As part of the phase I metabolism, the primary metabolites are the 10-N-glucuronide and the 4′-N-desmethyl olanzapine, which are clinically inactive and formed by the activity of CYP1A2. On the other hand, CYP2D6 catalyzes the formation of 2-OH olanzapine, and the flavin-containing monooxygenase (FMO3) is responsible for N-oxide olanzapine (Yang et al., 2017).

The results obtained in this study show that EC systems can mimic these metabolic reactions and, in some cases, produce the same metabolites observed in conventional enzymatic systems that exploit CYP450 enzymes. However, no previous reports directly compared the similarity of metabolic profiles between simulation systems and traditional *in vitro* metabolism systems with real patient samples. To address this gap, a metabolism simulation system using EC/MS were developed and demonstrated its ability to closely mimic the metabolite profiles produced by traditional microsomal incubation systems. All the postulated phase I and II metabolic reactions are typical of processes catalyzed by isoenzymes of the cytochrome P450 group. It may be concluded that the liver microsomes having enzymatic activity may be very useful in the study of the metabolism of antidepressants drugs. During the study of oxidation processes, the formation of potential metabolites was observed, consistent with the literature data and those formed by oxidation using cytochrome P450 isoenzymes present in the microsomal fraction of liver cells. Based on the obtained results, it can be stated that this method can be effectively used to predict oxidation processes initiated by single-electron oxidation, such as N-dealkylation, S-oxidation, P-oxidation, the oxidation of alcohols, the hydroxylation of aromatic systems and dehydrogenation. It is not beneficial in the case of reactions initiated by direct acquisition of a hydrogen atom, such as O-dealkylation or aliphatic hydroxylation of unsubstituted aromatic rings, due to the excessively high oxidation potential required for electrochemical oxidation reactions.

## 5 Conclusion

Electrochemistry in combination with mass spectrometry (MS) creates a powerful platform for the simulation of various oxidation and reduction processes in life sciences. It is a complementary technique to traditional *in vivo* or *in vitro* metabolism studies, and delivers the oxidative metabolic fingerprint of a molecule in a very short time. The combination of EC-MS was applied for the *in vitro* determination of studied psychotropic drugs and their potential metabolites./MS experiments were performed to structure the identification of the detected potential metabolites,. Appropriate conditions for oxidation and identification processes such parameters as the potential value, mobile phase (type and pH) working electrode and a flow rate of mobile phase were optimized. The performed experiments consisted of two stages: the electrochemical oxidation of the analyzed samples (phase I metabolic transformation), the addition of glutathione GSH for and follow-up reactions (phase II conjunction). The electrochemical results were compared with two methods: *in vivo* experiments where whole blood samples from patients after psychotropic drugs were administered and *in vitro* experiments by analyzing metabolism of selected drugs against metabolizing enzymes present in the microsomal fraction of liver cells. The obtained results prove that, identified electrochemical products are reflected in the potential metabolites for microsomal enzyme samples and real samples from patients. It testifies that the combination of electrochemistry and mass spectrometry is an effective method of drug metabolism simulation. Further development of this technique will probably allow it to be used as an alternative *in vitro* method to study oxidation-reduction reactions. Moreover, central composite design (CCD) could be successfully used for the selection of MS parameters for the studied psychotropic drugs as well as their pharmacologically active metabolites (MRM transitions). The oxidation process of studied psychotropic drugs in the electrochemical system is influenced by the buffer’s type, concentration, pH, and the type of working electrode used. The EC-ESI-MS technique gives the possibility to simulate the psychotropic drugs metabolism (pH > seven mainly for dehydrogenation; pH 7 N-dealkylation, hydroxylation; pH < 7 S-oxidation; MD mainly for hydroxylation, N-dealkylation; GC for dehydrogenation; Pt for N-dealkylation; Au for hydroxylation). Performed in controlled conditions, an *in vitro* study on metabolic changes of selected psychotropic drugs using human liver microsomes allows them to predict the potential pathways of transformation in the patient’s body. The analysis of plasma samples from patients confirmed the possibility of the identification of some products of oxidation processes using EC-ESI-MS. The electrochemistry-mass spectrometry (EC-MS) approach generated potential metabolites from selected psychotropic drugs. EC-MS technique provides an analytical tool to simulate phase I and II metabolism for each target compound, making it one of the future alternative methods for *in vitro* studies. EC-MS was revealed to be a suitable analytical tool to procure a feasible analytical base for the envisioned *in vivo* experiments.

## Data Availability

The raw data supporting the conclusions of this article will be made available by the authors, without undue reservation.
