# Supplementary material for: Electrochemical simulation of psychotropic drug metabolism compared to in vivo processes using liquid chromatography and mass spectrometry
Source: Front Pharmacol. 2025 Aug 28;16:1637852. doi: 10.3389/fphar.2025.1637852 (PMC12422924; doi:10.3389/fphar.2025.1637852)
Supplement: Supplementary file 1 [file Supplementaryfile1.docx]

**SUPPLEMENTARY INFORMATION**

**Electrochemical simulation of psychotropic drug metabolism compared to *in vivo* processes using liquid chromatography and mass spectrometry**

Table S1. Metabolism simulation analysis conditions for selected psychotropic drugs.

| **Working electrode** | **Potential range** | **Phase I simulation** | **Phase II simulation** | **Flow rate** | **Reaction cell** |
| --- | --- | --- | --- | --- | --- |
| GC | 0 – 2000 mV | Mobile phase (MP): 5-10 μM drug +  10 mM ammonium formate (pH=3,5±0,3) + 25% ACN (1:1 *v/v*)  or  5-10 μM drug +  10 mM r-r ammonium acetate (pH=5,7 i 9) + 25% ACN (1:1 *v/v*) | FR + 25 μM GSH | 10 μl/min | ReactorCell^TM^ |
| Pt | 0 – 2000 mV |  |  |  |  |
| Au | 0 – 2000 mV |  |  |  |  |
| MD | 0 – 3000 mV |  |  |  |  |

## **Central composite design (CCD)**

At the stage of selecting the mass spectrometer operating parameters for the tested analytes, a central composie design (CCD) was used, significantly facilitating the planning of the experiment. The drying gas temperature was tested in the range of 290-350̊C, while the fragmentor voltage was 70 to 150 V. The plan consisted of 9 experiments (Table S2), and the parameter values ​​were normalized as -1, 0, and +1.

**Table S2.** CCD for MS parameters selection.

| Input variables | Level | | |
| --- | --- | --- | --- |
|  | Low (-1) | Medium (0) | High (+1) |
| (*X_1_*) Voltage on the fragmentor [V] | 70 | 110 | 150 |
| (*X_2_*) Drying gas temperature [̊C] | 290 | 320 | 350 |
| Experiments number | *X_1_* | *X_2_* |  |
| 5 | 290 (-1) | 110 (+1) |  |
| 1 | 350 (+1) | 110 (-1) |  |
| 4 | 320 (0) | 110 (0) |  |
| 8 | 290 (0) | 150 (+1) |  |
| 3 | 290 (0) | 70 (-1) |  |
| 7 | 350 (-1) | 150 (-1) |  |
| 9 | 320 (-1) | 150 (0) |  |
| 2 | 320 (+1) | 70 (0) |  |
| 6 | 350 (+1) | 70 (+1) |  |

The results are presented graphically in spatial response surface plots for CLO, RIS, and VEN (Figure S1). The determined values ​​of drying gas temperature and fragmentor voltage are presented in Table 2.


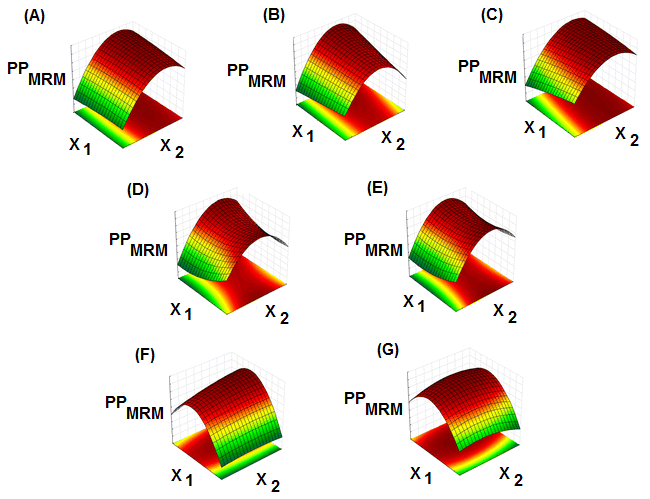


**Figure S1.** Response surface area for the output quantity (PP_MRM_) as a function of the fragmentor voltage (X1) and drying gas temperature (X2) for (A) CLO, (B) DCLO, (C) NoxCLO, (D) RIS, (E) HRIS, (F) VEN, and (G) DVEN.

The graphs show that for clozapine and its metabolites, the best fit for conditions depends on X2. The higher the drying gas value, the better the fit of the system and the larger the surface area. The value of X1, i.e. the voltage on the fragmentor, is not important here. The results for risperidone and its metabolite are similar. However, for venlafaxine and its metabolite, it is the other way around. The fit depends on the voltage on the fragmentor. Initially, the higher the value of X1, the better the fit, but after reaching the optimal value determining the best fit, further increase in X1 causes the result to deteriorate. However, the value of the drying gas is not significant here. The conclusion is that with the same parameters of drying gas temperature and voltage on the fragmentor for the drug, their metabolites can be determined and identified simultaneously.

Conditioning the bed:

approx. 20 µl of solvent, which is used later for the subsequent elution of analytes

Rinsing the sorbent bed:

After emptying the syringe, it is rinsed with a small volume of water

Sample introduction:

by repeated movement of the piston, 10-1000 µl of liquid sample can be passed through the sorbent bed in the syringe

Washing the sorbent bed:

The sorbent bed should be washed to remove interfering substances

Drying the sorbent bed

Elution of analytes using an organic solvent

**Figure S2.** Flow chart of the analytical procedure using the MEPS technique.

**Table S3.** Properties of the tested MEPS sorbents.

| **Type of stationary phase** | **Abbreviated name** | **Surface area [m^2^/g]** | **Grain diameter [μm]** | **Pore size [Å]** | **Sorption capacity** | **Carbon content [%]** | **pH stability** |
| --- | --- | --- | --- | --- | --- | --- | --- |
| *Unmodified silica* | SIL | 500 | 45 | 60 | 120-200 μg | - | 2-9 |
| *Diol* | C_2_ |  |  |  |  | 4 |  |
| *Octyl* | C_8_ |  |  |  |  | 8 |  |
| *Strong cation exchange* | SCX |  |  |  | 0,002 meq | - |  |
| *Octadecyl* | C_18_ |  |  |  | 120-200 μg | 14 |  |

**Table S4.** Characteristics of the group of isoenzymes included in CYP450.

| **Enzyme** | **Enzymatic activity[pmol/(mg×min)]** |
| --- | --- |
| *All P450* | 250 pmole/mg |
| *Cyt. b_5_* | 460 pmole/mg |
| *CYP1A2* | 820 |
| *CYP2A6* | 1200 |
| *CYP2B6* | 28 |
| *CYP2C8* | 190 |
| *CYP2C9* | 3000 |
| *CYP2C19* | 53 |
| *CYP2D6* | 99 |
| *CYP2E1* | 1900 |
| *CYP3A4* | 4900 |
| *CYP4A11* | 3500 |
| *UGT1A1* | 920 |
| *UGT1A4* | 890 |
| *UGT1A9* | 2400 |

**Determination of validation parameters of developed analytical procedures**

Universally used validation procedures were used to ensure the highest possible reliability of the results obtained during the conducted studies. Among the recommended validation parameters characterizing analytical methods, the following were determined in the studies: selectivity, linearity, limit of detection and quantification, accuracy, repeatability, precision, stability of the analyte in the biological matrix, recovery. When selecting the parameters included in the validation process, the nature of the conducted studies and the requirements imposed on them were primarily guided by the nature of the conducted studies.

*Selectivity of method*

An important element of method validation is to determine its selectivity, as the ability to determine a specific analyte in the presence of other components of the sample matrix, under given analysis conditions. The selectivity of the methods developed in this thesis was determined based on the results of determining biological samples from 5 individuals who were not taking any medications. Plasma and biological samples containing various endogenous compounds that may affect the measurement of the analyte content were prepared according to the procedures described in the experimental part. Additionally, plasma extracts enriched with various amounts of the compounds being determined were analyzed. The evaluation of the results consisted in determining the extent to which the substances present in the tested sample affect the determination of the analytes. The combination of liquid chromatography with a mass spectrometer additionally increased the selectivity of the determination. In the case of the LC-ESI-MS technique, a reduction in the number of additional peaks that hinder the interpretation of the chromatograms was observed. In addition, the reduction of the background in the chromatograms and, consequently, the increase of the signal-to-noise ratio allowed the detection of the tested compounds at lower concentrations, which consequently significantly reduced their detection and quantification limits. The probability of incorrect identification in the MS/MS analysis was negligible, because the transitions of primary ions to secondary ions were scanned, i.e. pairs of ions corresponding to the double fragmentation reactions characteristic of a given molecule, and no single ions (SIM). Moreover, the studies found that the use of the analysis mode recording selected pairs of MRM ions of the tested compounds eliminates the need for additional sample purification using microextraction on a packed sorbent (MEPS).

*Linearity range of methods, limits of quantification and detection*

Chromatographic techniques are based on relative measurement, which consists in comparing the detector signal obtained for the analyte present in the reference sample, with precisely known concentration, and in the test sample. A stock solution with a concentration of 1 mg/ml was used to prepare a series of working solutions. Appropriate amounts of working solutions of analytes were added to plasma samples in which the compounds being determined had not been previously detected. The parameters that play an important role in the process of validating the analytical method are the limits of quantification (LOD) and detection (LOQ). The limits of detection were determined based on the value of the standard deviation of the free term of the calibration curve and the slope angle of the curve. The limits of quantification were calculated as three times the LOD value. The validation parameters obtained for individual analytes are listed in **Table S5**.

**Table S5.** Linearity ranges, calibration curve parameters and LOD and LOQ of the tested compounds.

| **Compound** | **Linearity (ng/ml)** | **Regression equation** | **R2** | **LOD (ng/ml)** | **LOQ (ng/ml)** |
| --- | --- | --- | --- | --- | --- |
| QUE | 1-500 | y = 18,98x – 7,577 | 0.9993 | 0.031 | 0.093 |
| CLO | 1-500 | y = 41,83x + 45,4 | 0.9989 | 0.034 | 0.102 |
| ARI | 1-500 | y = 157,5x + 206,9 | 0.9944 | 0.028 | 0.084 |
| VEN | 1-500 | y = 37,69x – 12,80 | 0.9989 | 0.056 | 0.168 |
| VOR | 1-500 | y = 19,39x – 8,617 | 0.9973 | 0.029 | 0.087 |

The developed LC-MS/MS method was linear in the concentration range from 1 ng/ml to 500 ng/ml. Linearity in the above concentration ranges was confirmed by assessing the coefficients of determination, which were high for all calibration curves (R2>0.9830). Regression equations were determined for the analytes, while coefficients of variation (CV) were estimated for the components of the regression equations, which are in the ranges: CV (Sa) - 0.41-0.92%; CV (Sb) - 0.96-1.43% and CV (Sxy) - 1.68-2.37%. The limits of detection and quantification were calculated in the determined range of the method linearity for each of the compounds tested (**Table S5**).

*Repeatability, precision and accuracy of method*

In order to estimate the repeatability of the methods, the coefficient of variation CV was calculated for three series of samples with different concentrations (LQC, MQC, HQC), to which a specific amount of the standard of a given compound was added. Different concentrations of analytes were used to determine repeatability. The concentrations appropriate for the tested level of LQC, MQC and HQC were selected from the concentration range within the range of applicability of the method, and their values ​​for individual analytes are given in **Table S6**. Each batch consisted of 3 independently prepared samples, which were analyzed within one day. Precision was also determined, which allowed for determining the long-term deviation of the measurement process. The precision value was calculated as the total coefficient of variation for all results obtained based on analyses conducted over five consecutive days. The obtained results are given in **Table S6**.

The accuracy of the developed drug determination methods was checked by analyzing plasma extracts enriched in individual compounds. Using the obtained areas under the peak and the prepared calibration curves, the concentrations of analytes were calculated, and then the relative error. Accuracy was calculated for both results obtained based on analyses conducted over one day and over five consecutive days. The obtained results are given in **Table S6**.

The coefficients of variation obtained for the LC-MS/MS method for each of the three concentrations of individual analytes range from 0.58% to 4.19% (**Table S6**). The values ​​of the coefficients of variation obtained based on analyses conducted over one day (repeatability) and over five days (intermediate precision) are at the same level. The developed method is also characterized by high accuracy (-5.26%<RE<5.01%).

**Table S6.** Repeatability, precision, accuracy and recoveries of determined compounds.

| **Compound** | **Amount added [ng/ml]** | **Repeatability and accuracy (determined within 1 day)** | | | **Intermediate precision and accuracy (determined within 5 days)** | | | **Recovery [%]** | **ME [%]** |
| --- | --- | --- | --- | --- | --- | --- | --- | --- | --- |
|  |  | **Amount measured**  **[ng/ml]** | **CV [%]** | **RE [%]** | **Amount measured**  **[ng/ml]** | **CV [%]** | **RE [%]** |  |  |
| QUE | 1 | 0.98 | 3.98 | -2.04 | 1.03 | 4.11 | 2.91 | 83.43 | 8.16 |
|  | 150 | 149.45 | 3.77 | -3.81 | 149.97 | 3.90 | -0.20 | 80.37 | -0.19 |
|  | 500 | 499.56 | 0.58 | -0.89 | 500.87 | 2.98 | 1.71 | 85.23 | 2.48 |
| CLO | 1 | 0.99 | 0.76 | -1.01 | 1.03 | 1.78 | 2.91 | 75.89 | -5.64 |
|  | 150 | 150.03 | 2.87 | 0.20 | 150.04 | 0.98 | 0.27 | 78.68 | -3.69 |
|  | 500 | 500.05 | 2.73 | 0.10 | 500.09 | 1.34 | 0.18 | 77.42 | 0.18 |
| ARI | 1 | 0.96 | 2.06 | -4.17 | 1.03 | 3.22 | 2.91 | 82.01 | 5.89 |
|  | 150 | 150.03 | 2.02 | 0.20 | 149.98 | 2.97 | -0.13 | 85.63 | 5.08 |
|  | 500 | 499.06 | 5.01 | -1.92 | 499.05 | 3.02 | -1.94 | 85.37 | 2.67 |
| VEN | 1 | 1.04 | 2.35 | 3.85 | 0.97 | 2.92 | -3.09 | 80.07 | 8.52 |
|  | 150 | 149.59 | 3.11 | -2.81 | 150.06 | 1.78 | 0.40 | 79.69 | 5.35 |
|  | 500 | 500.08 | 2.89 | 0.16 | 500.15 | 0.79 | 0.30 | 79.85 | 4.02 |
| VOR | 1 | 0.98 | 2.78 | -2.04 | 0.94 | 2.92 | -6.38 | 83.32 | -9.06 |
|  | 150 | 149.68 | 1.96 | -2.18 | 150.06 | 3.16 | 0.40 | 82.15 | -8.36 |
|  | 500 | 500.06 | 2.05 | 0.12 | 500.11 | 2.98 | 0.22 | 80.15 | -7.74 |

*Recovery of analytes, matrix effect*

The recovery degree test was carried out by adding three different amounts of standards of each of the drugs to be determined to the samples. Hence, the spiked samples contained analytes at the same concentration levels (LQC, MQC, HQC) as used to determine the remaining validation parameters. The matrix effect was assessed for three concentration levels of a given analyte in the samples (LQC, MQC and HQC). The ME values ​​are listed in the table. Based on them, it can be concluded that matrix effects result in a slight weakening or strengthening of the signal (ME ranging from -9.06% to 12.43%) originating from the analyte.

*Stability of selected drugs*

The stability test was carried out by adding three different amounts of standards of each of the drugs to the samples. Hence, the spiked samples contained the analytes at the same concentration levels (LQC, MQC, HQC) as used to determine the remaining validation parameters. Samples with the appropriate standard solution added were stored at appropriate temperature conditions and for a specified period of time: 24 hours in an automatic sampler (5°C), 12 hours at room temperature, 30 days at -20°C and three freeze/thaw cycles. **Table S7** presents the results obtained using the LC-MS/MS method.

**Table S7.** Stability in biological samples depending on the sample storage conditions used.

| **Compound** | **Auto-sampler** | | **Short-term stability** | | **Long-term stability** | | **Three cycles** | |
| --- | --- | --- | --- | --- | --- | --- | --- | --- |
|  | **Amount measured**  **[ng/ml]** | **RE [%]** | **Amount measured**  **[ng/ml]** | **RE [%]** | **Amount measured**  **[ng/ml]** | **RE [%]** | **Amount measured**  **[ng/ml]** | **RE [%]** |
| QUE | 150.098 | 0.64 | 150.075 | 0.50 | 149.829 | -1.15 | 150.637 | 4.07 |
| CLO | 150.166 | 1.09 | 150.189 | 1.24 | 150.092 | 0.61 | 150.829 | 5.24 |
| ARI | 149.904 | -0.64 | 149.944 | -0.37 | 149.972 | -0.19 | 149.627 | -2.55 |
| VEN | 149.504 | -3.42 | 149.609 | -2.68 | 150.082 | 0.54 | 149.292 | -4.95 |
| VOR | 149.609 | -2.68 | 149.905 | -0.64 | 149.936 | -0.43 | 150.974 | 6.10 |


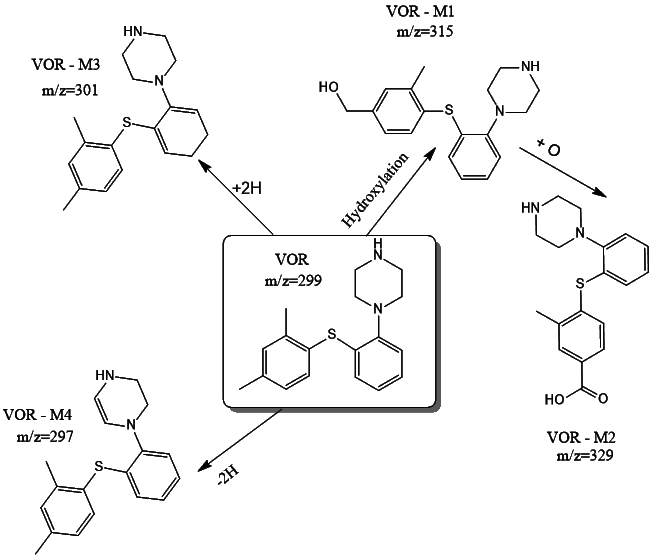


**Figure S3.** Proposed pathway of the electrochemical processes for vortioxetine (MD electrode, pH=7).


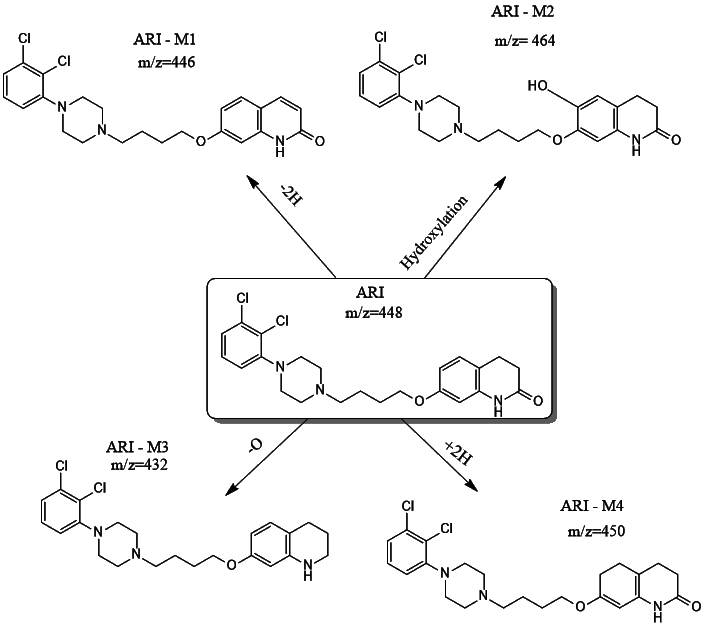


**Figure S4.** Proposed pathway of the electrochemical processes for aripiprazole (MD electrode, pH=7).


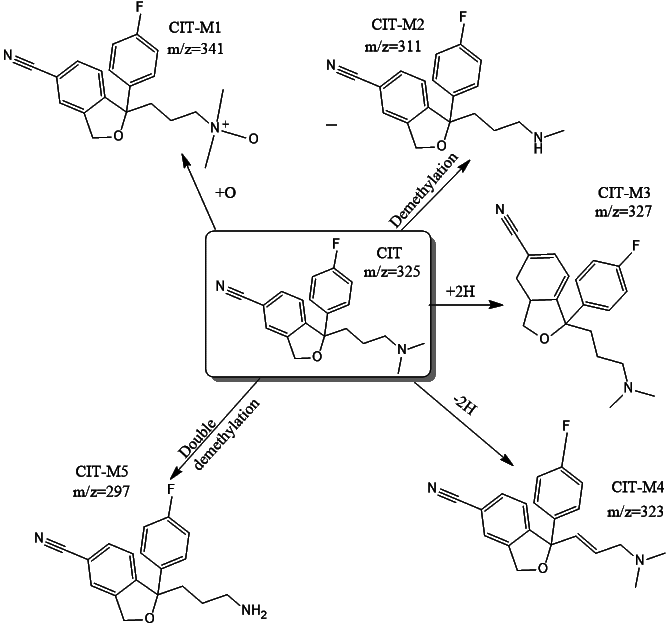


**Figure S5.** Proposed pathway of the electrochemical processes for citalopram (MD electrode, pH=7).


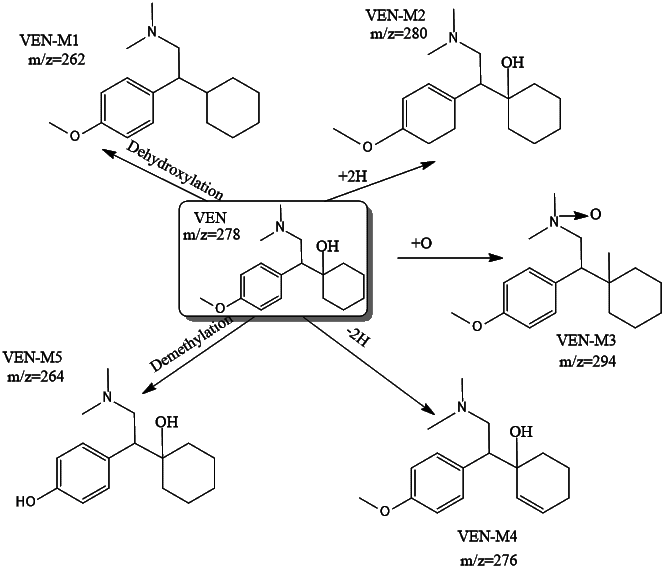


**Figure S6.** Proposed pathway of the electrochemical processes for venlafaxine.


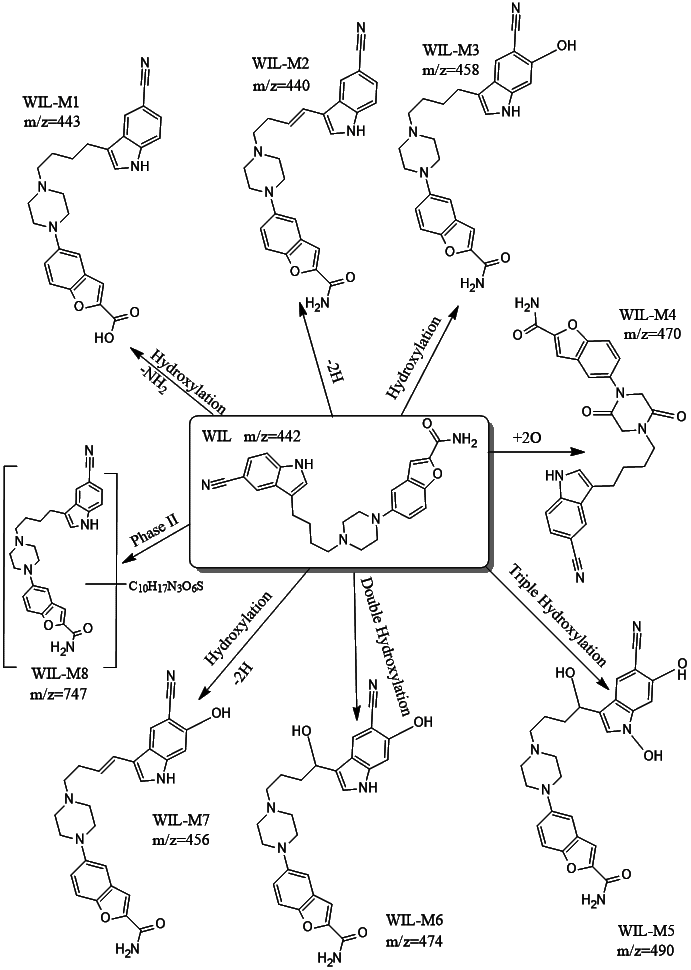


**Figure S7.** Proposed pathway of the electrochemical processes for vilazodone.


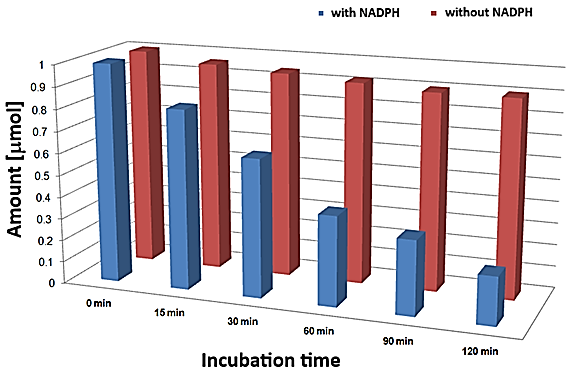


**Figure S8.** Effect of the presence of the enzyme reaction cofactor-NADPH on the transformation of a psychotropic drug (clozapine).

**Fragmentation spectra of psychotropic drugs and their metabolites**

Fragmentation mass spectra can provide much information about the structure of the compound being studied. Below, Figure S9 presents probable fragmentation paths of the psychotropic drugs studied, and Figure S10 for their metabolites.

1. **
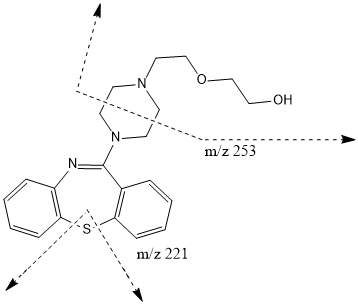

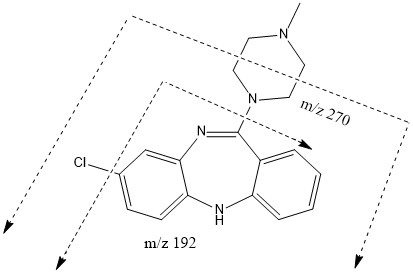
 b)**

**
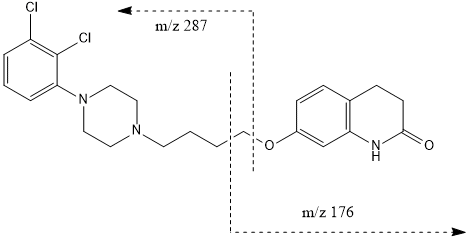

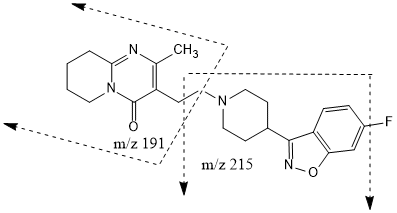
b) d)**

**
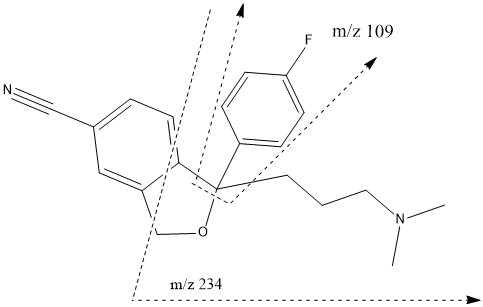

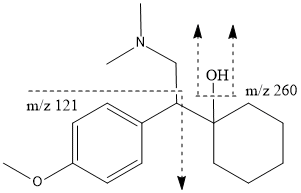
e) f)**

**
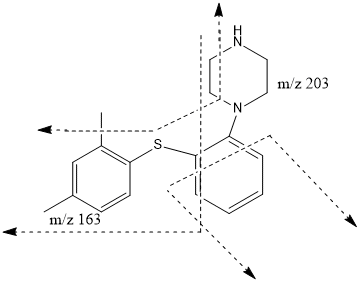
**

**
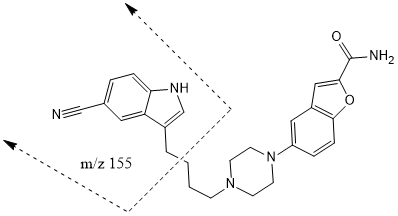
g) h)**

**Figure S9.** Proposed fragmentation pathway for psychotropic drugs: a) QUE, b) CLO, c) ARP, d) RIS, e) CIT, f) VEN, g) WIL, h) VOR.

**
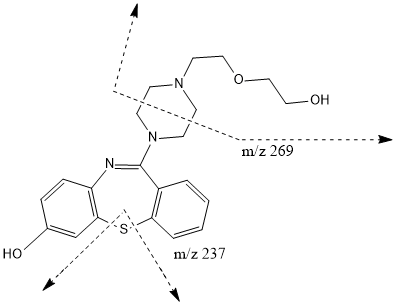

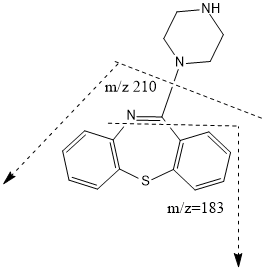
a) b)**


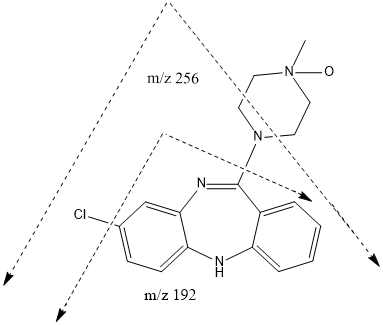


**
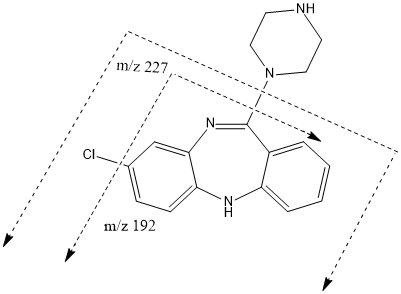
c) d)**

**
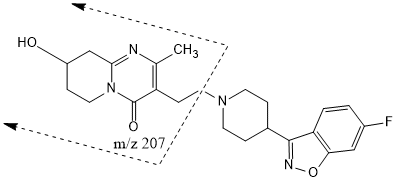

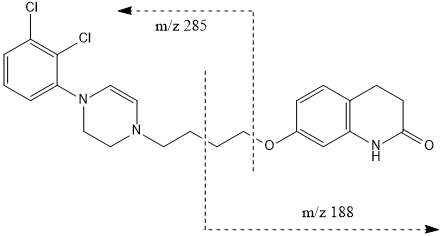
e) f)**

**
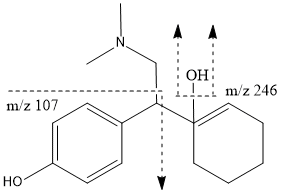

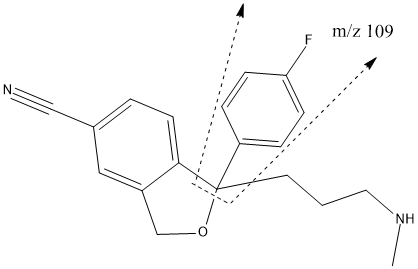
g) h)**

**Figure S10.** Proposed fragmentation pathway for selected metabolites of psychotropic drugs: a) 7-hydroxyquetiapine, b) norquetiapine, c) clozapine-N-oxaid,

d) N-desmethylclozapine, e) dehydroaripiprazole, f) 9-hydroxyrisperidone,

g) N-desmethylcitalopram, h) O-desmethylvenlafaxine.

**Table S8.** In silico predicted sites of the metabolism of the target compounds (FAME3) software).


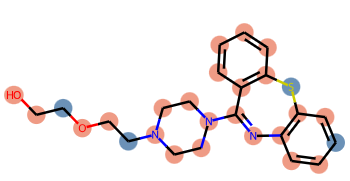
 Quetiapine

| **Atom** | **Probability** | **SOM** | **Fame score** |
| --- | --- | --- | --- |
| N.1 | 0.04 | No | 0.84 |
| C.2 | 0.04 | No | 0.79 |
| C.3 | 0.00 | No | 0.84 |
| C.4 | 0.00 | No | 0.87 |
| S.5 | 0.74 | Yes | 0.84 |
| C.6 | 0.01 | No | 0.84 |
| C.7 | 0.01 | No | 0.87 |
| C.8 | 0.03 | No | 0.87 |
| C.9 | 0.01 | No | 0.87 |
| C.10 | 0.69 | Yes | 0.84 |
| C.11 | 0.04 | No | 0.83 |
| C.12 | 0.01 | No | 0.87 |
| C.13 | 0.05 | No | 0.87 |
| C.14 | 0.04 | No | 0.82 |
| C.15 | 0.00 | No | 0.81 |
| N.16 | 0.06 | No | 0.78 |
| C.17 | 0.04 | No | 0.92 |
| C.18 | 0.07 | No | 0.92 |
| N.19 | 0.06 | No | 0.78 |
| C.20 | 0.69 | Yes | 0.77 |
| C.21 | 0.08 | No | 0.70 |
| O.22 | 0.02 | No | 0.70 |
| C.23 | 0.72 | Yes | 0.67 |
| C.24 | 0.09 | No | 0.70 |
| O.25 | 0.20 | No | 0.76 |
| C.26 | 0.07 | No | 0.92 |
| C.27 | 0.04 | No | 0.92 |


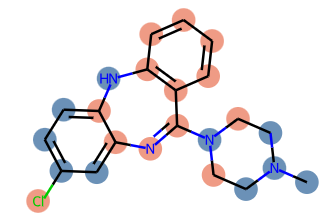
 Clozapine

| **Atom** | **Probability** | **SOM** | **Fame score** |
| --- | --- | --- | --- |
| C.1 | 0.73 | Yes | 0.76 |
| N.2 | 0.87 | Yes | 0.78 |
| C.3 | 0.65 | Yes | 0.92 |
| C.4 | 0.06 | No | 0.92 |
| N.5 | 0.66 | Yes | 0.80 |
| C.6 | 0.06 | No | 0.92 |
| C.7 | 0.65 | Yes | 0.92 |
| C.8 | 0.03 | No | 0.80 |
| N.9 | 0.02 | No | 0.81 |
| C.10 | 0.00 | No | 0.84 |
| C.11 | 0.68 | Yes | 0.86 |
| C.12 | 0.65 | Yes | 0.86 |
| C.13 | 0.70 | Yes | 0.86 |
| C.14 | 0.71 | Yes | 0.83 |
| C.15 | 0.01 | No | 0.82 |
| N.16 | 0.71 | Yes | 0.82 |
| C.17 | 0.00 | No | 0.84 |
| C.18 | 0.00 | No | 0.81 |
| C.19 | 0.01 | No | 0.81 |
| C.20 | 0.03 | No | 0.82 |
| C.21 | 0.04 | No | 0.85 |
| C.22 | 0.03 | No | 0.85 |
| Cl.23 | 0.00 | No | 0.74 |


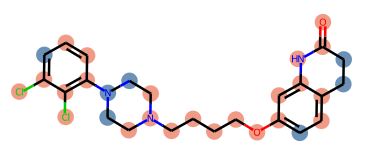
 Aripiprazole

| **Atom** | **Probability** | **SOM** | **Fame score** |
| --- | --- | --- | --- |
| Cl.1 | 0.00 | No | 0.96 |
| C.2 | 0.07 | No | 0.90 |
| C.3 | 0.78 | Yes | 0.96 |
| C.4 | 0.04 | No | 0.90 |
| C.5 | 0.08 | No | 0.85 |
| C.6 | 0.07 | No | 0.74 |
| N.7 | 0.72 | Yes | 0.72 |
| C.8 | 0.74 | Yes | 0.88 |
| C.9 | 0.08 | No | 0.88 |
| N.10 | 0.07 | No | 0.69 |
| C.11 | 0.15 | No | 0.73 |
| C.12 | 0.01 | No | 0.70 |
| C.13 | 0.02 | No | 0.72 |
| C.14 | 0.08 | No | 0.76 |
| O.15 | 0.04 | No | 0.73 |
| C.16 | 0.00 | No | 0.84 |
| C.17 | 0.64 | Yes | 0.86 |
| C.18 | 0.00 | No | 0.83 |
| C.19 | 0.00 | No | 0.83 |
| C.20 | 0.00 | No | 0.81 |
| N.21 | 0.04 | No | 0.74 |
| C.22 | 0.13 | No | 0.81 |
| O.23 | 0.00 | No | 0.85 |
| C.24 | 0.73 | Yes | 0.83 |
| C.25 | 0.71 | Yes | 0.77 |
| C.26 | 0.05 | No | 0.83 |
| C.27 | 0.08 | No | 0.88 |
| C.28 | 0.74 | Yes | 0.88 |
| C.29 | 0.00 | No | 0.85 |
| Cl.30 | 0.02 | No | 0.81 |


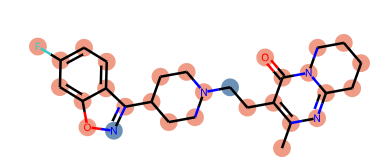
 Risperidone

| **Atom** | **Probability** | **SOM** | **Fame score** |
| --- | --- | --- | --- |
| C.1 | 0.10 | No | 0.68 |
| C.2 | 0.06 | No | 0.68 |
| C.3 | 0.04 | No | 0.63 |
| C.4 | 0.02 | No | 0.68 |
| O.5 | 0.02 | No | 0.70 |
| N.6 | 0.06 | No | 0.72 |
| C.7 | 0.13 | No | 0.72 |
| N.8 | 0.07 | No | 0.74 |
| C.9 | 0.37 | No | 0.72 |
| C.10 | 0.35 | No | 0.77 |
| C.11 | 0.30 | No | 0.70 |
| C.12 | 0.21 | No | 0.73 |
| C.13 | 0.02 | No | 0.66 |
| C.14 | 0.72 | Yes | 0.67 |
| N.15 | 0.06 | No | 0.70 |
| C.16 | 0.30 | No | 0.83 |
| C.17 | 0.01 | No | 0.83 |
| C.18 | 0.05 | No | 0.67 |
| C.19 | 0.01 | No | 0.83 |
| C.20 | 0.30 | No | 0.83 |
| C.21 | 0.12 | No | 0.69 |
| C.22 | 0.02 | No | 0.72 |
| C.23 | 0.04 | No | 0.77 |
| C.24 | 0.02 | No | 0.80 |
| C.25 | 0.06 | No | 0.83 |
| C.26 | 0.02 | No | 0.81 |
| C.27 | 0.03 | No | 0.75 |
| O.28 | 0.39 | No | 0.75 |
| N.29 | 0.57 | Yes | 0.72 |
| F.30 | 0.00 | No | 0.75 |


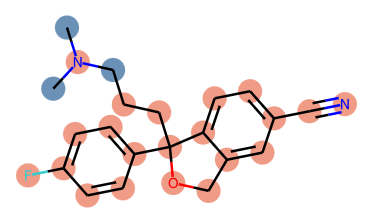
 Citalopram

| **Atom** | **Probability** | **SOM** | **Fame score** |
| --- | --- | --- | --- |
| F.1 | 0.00 | No | 0.99 |
| C.2 | 0.00 | No | 0.99 |
| C.3 | 0.00 | No | 1.00 |
| C.4 | 0.00 | No | 1.00 |
| C.5 | 0.00 | No | 0.99 |
| C.6 | 0.00 | No | 1.00 |
| C.7 | 0.00 | No | 1.00 |
| C.8 | 0.00 | No | 0.99 |
| O.9 | 0.00 | No | 0.99 |
| C.10 | 0.00 | No | 0.99 |
| C.11 | 0.00 | No | 0.99 |
| C.12 | 0.00 | No | 0.99 |
| C.13 | 0.00 | No | 0.99 |
| C.14 | 0.01 | No | 0.99 |
| N.15 | 0.00 | No | 1.00 |
| C.16 | 0.00 | No | 0.99 |
| C.17 | 0.00 | No | 0.99 |
| C.18 | 0.00 | No | 0.99 |
| C.19 | 0.00 | No | 0.99 |
| C.20 | 0.00 | No | 0.99 |
| C.21 | 0.91 | Yes | 0.99 |
| N.22 | 0.02 | No | 0.99 |
| C.23 | 0.97 | Yes | 1.00 |
| C.24 | 0.97 | Yes | 1.00 |


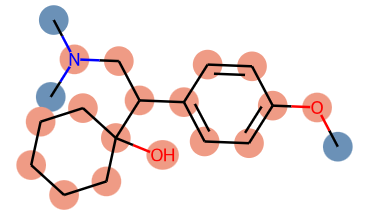
 Venlafaxine

| **Atom** | **Probability** | **SOM** | **Fame score** |
| --- | --- | --- | --- |
| O.1 | 0.32 | No | 0.66 |
| C.2 | 0.11 | No | 0.68 |
| C.3 | 0.05 | No | 0.72 |
| C.4 | 0.01 | No | 0.85 |
| C.5 | 0.03 | No | 0.91 |
| C.6 | 0.06 | No | 0.85 |
| C.7 | 0.00 | No | 0.69 |
| O.8 | 0.04 | No | 0.69 |
| C.9 | 0.64 | Yes | 0.73 |
| C.10 | 0.06 | No | 0.85 |
| C.11 | 0.03 | No | 0.91 |
| C.12 | 0.22 | No | 0.73 |
| N.13 | 0.18 | No | 0.71 |
| C.14 | 0.44 | Yes | 0.71 |
| C.15 | 0.44 | Yes | 0.71 |
| C.16 | 0.07 | No | 0.71 |
| C.17 | 0.17 | No | 0.69 |
| C.18 | 0.35 | No | 0.64 |
| C.19 | 0.17 | No | 0.69 |
| C.20 | 0.07 | No | 0.71 |


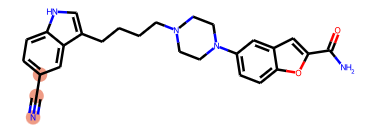
 Vilazodone

| **Atom** | **Probability** | **SOM** | **Fame score** |
| --- | --- | --- | --- |
| N.1 | 0.01 | No | 0.64 |
| C.2 | 0.09 | No | 0.62 |
| C.3 | 0.09 | No | 0.67 |


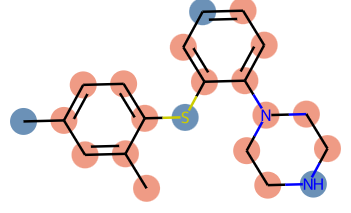
 Vortioxetine

| **Atom** | **Probability** | **SOM** | **Fame score** |
| --- | --- | --- | --- |
| C.1 | 0.16 | No | 0.81 |
| C.2 | 0.00 | No | 0.84 |
| C.3 | 0.02 | No | 0.86 |
| C.4 | 0.01 | No | 0.81 |
| C.5 | 0.80 | Yes | 0.74 |
| C.6 | 0.03 | No | 0.89 |
| C.7 | 0.03 | No | 0.85 |
| C.8 | 0.00 | No | 0.78 |
| S.9 | 0.69 | Yes | 0.74 |
| C.10 | 0.00 | No | 0.78 |
| C.11 | 0.01 | No | 0.83 |
| N.12 | 0.05 | No | 0.75 |
| C.13 | 0.10 | No | 0.89 |
| C.14 | 0.07 | No | 0.89 |
| N.15 | 0.76 | Yes | 0.73 |
| C.16 | 0.07 | No | 0.89 |
| C.17 | 0.10 | No | 0.89 |
| C.18 | 0.00 | No | 0.83 |
| C.19 | 0.03 | No | 0.81 |
| C.20 | 0.67 | Yes | 0.78 |
| C.21 | 0.01 | No | 0.78 |


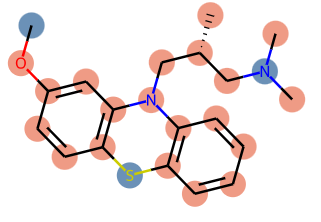
 Levomepromazine

| **Atom** | **Probability** | **SOM** | **Fame score** |
| --- | --- | --- | --- |
| O.1 | 0.11 | No | 0.71 |
| C.2 | 0.00 | No | 0.72 |
| C.3 | 0.01 | No | 0.72 |
| C.4 | 0.00 | No | 0.73 |
| N.5 | 0.12 | No | 0.70 |
| C.6 | 0.00 | No | 0.77 |
| C.7 | 0.00 | No | 0.76 |
| S.8 | 0.45 | Yes | 0.69 |
| C.9 | 0.00 | No | 0.71 |
| C.10 | 0.02 | No | 0.73 |
| C.11 | 0.20 | No | 0.75 |
| C.12 | 0.03 | No | 0.74 |
| C.13 | 0.07 | No | 0.75 |
| C.14 | 0.06 | No | 0.74 |
| C.15 | 0.01 | No | 0.74 |
| C.16 | 0.18 | No | 0.70 |
| C.17 | 0.04 | No | 0.70 |
| C.18 | 0.07 | No | 0.66 |
| C.19 | 0.14 | No | 0.72 |
| N.20 | 0.77 | Yes | 0.75 |
| C.21 | 0.32 | No | 0.80 |
| C.22 | 0.32 | No | 0.80 |
| C.23 | 0.77 | Yes | 0.76 |


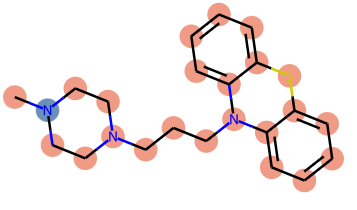
 Perazine

| **Atom** | **Probability** | **SOM** | **Fame score** |
| --- | --- | --- | --- |
| C.1 | 0.19 | No | 0.73 |
| N.2 | 0.74 | Yes | 0.72 |
| C.3 | 0.04 | No | 0.92 |
| C.4 | 0.06 | No | 0.84 |
| N.5 | 0.06 | No | 0.80 |
| C.6 | 0.06 | No | 0.84 |
| C.7 | 0.04 | No | 0.92 |
| C.8 | 0.12 | No | 0.78 |
| C.9 | 0.01 | No | 0.75 |
| C.10 | 0.04 | No | 0.75 |
| N.11 | 0.12 | No | 0.75 |
| C.12 | 0.00 | No | 0.82 |
| C.13 | 0.00 | No | 0.82 |
| C.14 | 0.01 | No | 0.83 |
| C.15 | 0.07 | No | 0.83 |
| C.16 | 0.01 | No | 0.83 |
| C.17 | 0.00 | No | 0.82 |
| S.18 | 0.24 | No | 0.75 |
| C.19 | 0.00 | No | 0.82 |
| C.20 | 0.01 | No | 0.83 |
| C.21 | 0.07 | No | 0.83 |
| C.22 | 0.01 | No | 0.83 |
| C.23 | 0.00 | No | 0.82 |
| C.24 | 0.00 | No | 0.82 |

Olanzapine

| **Atom** | **Probability** | **SOM** | **Fame score** |
| --- | --- | --- | --- |
| C.1 | 0.61 | Yes | 0.66 |
| N.2 | 0.88 | Yes | 0.66 |
| C.3 | 0.16 | No | 0.74 |
| C.4 | 0.09 | No | 0.73 |
| N.5 | 0.07 | No | 0.66 |
| C.6 | 0.09 | No | 0.73 |
| C.7 | 0.16 | No | 0.74 |
| C.8 | 0.04 | No | 0.67 |
| N.9 | 0.04 | No | 0.69 |
| C.10 | 0.00 | No | 0.77 |
| C.11 | 0.03 | No | 0.77 |
| C.12 | 0.16 | No | 0.76 |
| C.13 | 0.22 | No | 0.67 |
| C.14 | 0.07 | No | 0.66 |
| C.15 | 0.00 | No | 0.68 |
| N.16 | 0.14 | No | 0.69 |
| C.17 | 0.02 | No | 0.71 |
| S.18 | 0.11 | No | 0.75 |
| C.19 | 0.06 | No | 0.78 |
| C.20 | 0.12 | No | 0.66 |
| C.21 | 0.08 | No | 0.75 |
| C.22 | 0.01 | No | 0.80 |

**Table S9.** Recoveries of the analytes tested (c=10 ng/ml) using different MEPS-type sorbents and a mixture of ACN:MeOH:H_2_O (5:3:2; *v/v/v*).

| **Compound** | **SIL** | | **C2** | | **C8** | | **SCX** | | **C18** | |
| --- | --- | --- | --- | --- | --- | --- | --- | --- | --- | --- |
|  | **Recovery ±SD**  **(%±ng/ml)** | **CV (%)** | **Recovery ±SD**  **(%±ng/ml)** | **CV (%)** | **Recovery ±SD**  **(%±ng/ml)** | **CV (%)** | **Recovery ±SD**  **(%±ng/ml)** | **CV (%)** | **Recovery ±SD**  **(%±ng/ml)** | **CV (%)** |
| *QUE* | 26,98±0,024 | 1,64 | 35,89±0,016 | 3,19 | 63,27±0,008 | 2,50 | 15,1±0,027 | 1,26 | 95,48±0,029 | 3,38 |
| *HQUE* | 29,82±0,006 | 3,39 | 33,35±0,019 | 0,21 | 68,04±0,008 | 2,81 | 18,88±0,012 | 2,69 | 96,39±0,012 | 2,52 |
| *NQUE* | 31,97±0,009 | 1,12 | 32,41±0,017 | 0,08 | 69,93±0,009 | 0,40 | 13,10±0,023 | 1,80 | 93,29±0,025 | 2,57 |
| *CLO* | 24,92±0,012 | 2,66 | 39,77±0,009 | 3,58 | 68,66±0,005 | 0,71 | 18,43±0,013 | 1,55 | 95,94±0,005 | 0,9 |
| *NoxCLO* | 25,94±0,019 | 1,92 | 33,25±0,009 | 0,77 | 68,00±0,008 | 0,58 | 14,78±0,024 | 1,32 | 98,59±0,016 | 0,94 |
| *DCLO* | 30,37±0,008 | 2,21 | 32,40±0,005 | 1,16 | 68,81±0,020 | 3,60 | 14,88±0,021 | 1,38 | 99,71±0,027 | 3,17 |
| *ARI* | 22,45±0,015 | 0,53 | 38,37±0,003 | 1,74 | 65,89±0,016 | 1,10 | 18,30±0,025 | 1,44 | 99,11±0,020 | 1,33 |
| *DARI* | 20,92±0,023 | 2,67 | 32,42±0,027 | 2,94 | 63,35±0,019 | 2,81 | 17,13±0,011 | 0,08 | 93,27±0,008 | 2,81 |
| *RIS* | 24,49±0,049 | 1,80 | 48,10±0,023 | 2,50 | 62,41±0,017 | 2,57 | 15,94±0,019 | 1,92 | 98,04±0,008 | 2,50 |
| *HRIS* | 35,89±0,016 | 3,73 | 48,04±0,008 | 2,81 | 69,77±0,009 | 0,9 | 10,37±0,008 | 2,21 | 99,93±0,009 | 2,81 |
| *CIT* | 33,35±0,019 | 1,23 | 49,93±0,009 | 0,40 | 63,25±0,009 | 0,94 | 12,45±0,015 | 0,53 | 98,66±0,005 | 0,40 |
| *DCIT* | 32,41±0,017 | 2,93 | 48,66±0,005 | 0,71 | 62,40±0,005 | 3,17 | 10,92±0,023 | 2,67 | 98,00±0,008 | 0,71 |
| *VEN* | 39,77±0,009 | 2,11 | 48,00±0,008 | 0,58 | 68,37±0,003 | 1,33 | 14,49±0,049 | 1,80 | 98,81±0,020 | 0,58 |
| *DVEN* | 33,25±0,009 | 2,43 | 48,81±0,020 | 3,60 | 65,48±0,029 | 2,81 | 15,89±0,016 | 3,73 | 99,74±0,013 | 3,60 |
| *WIL* | 32,40±0,005 | 0,58 | 49,74±0,013 | 1,10 | 66,39±0,012 | 2,50 | 13,35±0,019 | 1,23 | 93,17±0,016 | 1,10 |
| *M10WIL* | 38,37±0,003 | 2,94 | 43,17±0,016 | 2,81 | 63,29±0,025 | 2,81 | 12,41±0,017 | 2,93 | 92,29±0,038 | 2,81 |
| *VOR* | 32,42±0,027 | 2,81 | 42,29±0,038 | 1,55 | 65,94±0,005 | 0,40 | 19,77±0,009 | 2,11 | 93,21±0,049 | 1,92 |
| *LEV* | 33,58±0,038 | 0,40 | 43,21±0,049 | 1,32 | 68,59±0,016 | 0,71 | 13,25±0,009 | 3,58 | 91,37±0,031 | 2,21 |
| *PER* | 31,30±0,017 | 0,71 | 41,37±0,031 | 1,38 | 69,71±0,027 | 1,16 | 12,40±0,005 | 0,77 | 92,40±0,005 | 0,53 |
| *OLA* | 35,20±0,092 | 0,58 | 39,47±0,038 | 1,44 | 69,11±0,020 | 1,74 | 18,37±0,003 | 1,16 | 98,37±0,003 | 2,67 |
| *DOLA* | 28,31±0,027 | 3,60 | 36,32±0,027 | 0,08 | 63,27±0,008 | 2,94 | 12,42±0,027 | 1,74 | 92,42±0,027 | 1,80 |
